# Daughter cell fate choice instructed preemptively by mother cells facing nutrient limitation

**DOI:** 10.1016/j.isci.2023.107198

**Published:** 2023-06-24

**Authors:** Dianpeng Zheng, Yaowen Mao, Yinglong Gao, Feng He, Jun Ma

**Affiliations:** 1 Women’s Hospital and Institute of Genetics, Zhejiang University School of Medicine, Hangzhou, Zhejiang 310058, China; 2 Zhejiang Provincial Key Laboratory of Genetic and Developmental Disorder, Hangzhou, Zhejiang 310058, China; 3 National Clinical Research Center for Child Health, Children’s Hospital, Zhejiang University School of Medicine, Hangzhou, Zhejiang 310052, China; 4 Women’s Reproductive Health Research Laboratory of Zhejiang Province, Hangzhou, Zhejiang 310006, China

**Keywords:** Biological sciences, Cell biology, Cell

## Abstract

Nutrients are vital to cellular activities, yet it is largely unknown how individual cells respond to nutrient deprivation. Live imaging results show that unlike the removal of amino acids or glutamine that immediately halts cell cycle progression, glucose withdrawal does not prevent cells from completing their current cycle. Although cells that begin to experience glucose withdrawal in S phase give rise to daughter cells with an equal choice of proliferation or quiescence, those enduring such experience in G1 phase give rise to daughter cells that predominantly enter quiescence. This fate choice difference stems from p21 protein accumulated during G2/M of the latter cells. Induced degradation of p21 permits daughter cells to enter S phase but with a consequent accumulation of DNA damage. These results suggest that mother cells that begin to experience glucose limitation in G1 phase take preemptive steps toward preventing daughter cells from making a harmful choice.

## Highlights

• Removal of amino acids, glutamine, or glucose impacts cell cycle progression differently
• Glucose removal in mother cells affects daughter cells’ proliferation-quiescence choice
• Daughter cells inherit p21 from mother cells experiencing glucose withdrawal in G1 phase
• Without glucose, p21 removal permits cells to enter S phase accumulating DNA damage

## Graphical abstract

**Figure:**
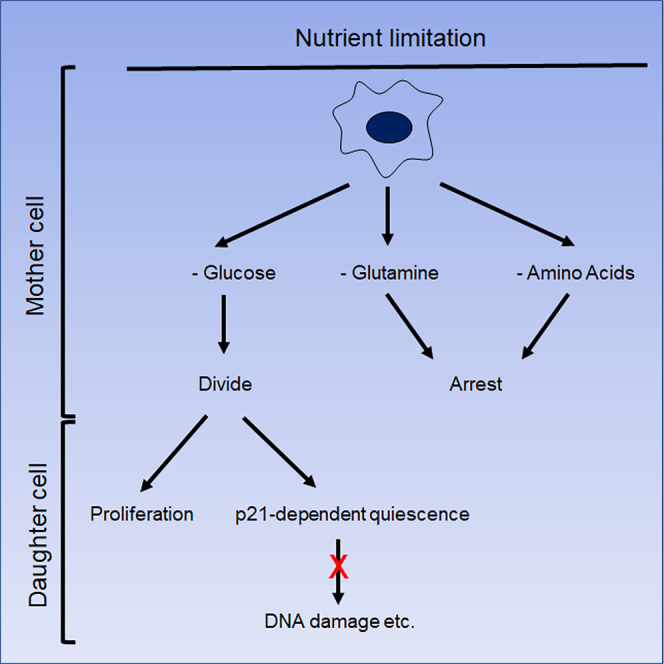

## Introduction

Proliferation is a deliberate decision that cells make relying on both intra- and extra-cellular inputs.^1^^,^^2^^,^^3^ One such input is the availability of nutrients, which provide sources for the production of energy and biomass necessary for cell growth and survival. For example, yeast cells make a decision before entering S phase, thus committing to genome duplication and cell division only when sufficient nutrients are available.^4^^,^^5^ Although yeast cells rely primarily on the availability of nutrients in such decision-making, mammalian cells have evolved to respond primarily to growth factors. This change is necessary for coordinating developmental and physiological processes that are unique to multicellular organisms. However, proliferation of mammalian cells cannot circumvent the need for nutrients. It is well documented that prolonged glucose limitation leads to cell-cycle arrest,^6^ but precisely how the input of glucose availability feeds into proliferative decision-making is not fully understood.

Recent studies suggest that unlike yeast cells, mammalian cells have two major restriction points, one located in G2/M phase (referred to as R1) and the other closer to the transition from G1 to S (referred to as R2).^5^^,^^7^ Although R1 represents a primary checkpoint that integrates inputs of growth signals and DNA damage signals, R2 has a location more similar to the yeast START point.^4^^,^^5^ It is thus possible that, as a nutrient signal, the glucose limitation signal may feed into cell cycle control through R2 to control G1/S transition as in yeast cells. Alternatively, or additionally, such a nutrient availability signal could be funneled into the cell cycle control through R1 with other inputs. Nutrient depletion is a special type of challenge that cells face because, without nutrients, cells will eventually run out of energy required for maintaining biological activities. It is thus unclear whether the halt in proliferation for cells facing glucose depletion is purely a passive consequence of a cell’s incapability or may actually be an action of protective value to cells under such conditions.

Much of our current knowledge of mammalian cell cycle control is derived from studies using synchronized or perturbed cells.^8^^,^^9^ These studies provide a general picture of the behavior of cell cycle control at the resolution of cell population. It is becoming increasingly clear that genetically identical cells in a population may exhibit a high degree of heterogeneity when responding to external inputs.^10^^,^^11^^,^^12^^,^^13^ Heterogeneity is a wide-spread phenomenon that is present even in a population of unperturbed cells with regard to their behavior in cell cycle progression.^14^^,^^15^^,^^16^^,^^17^ It is currently unclear how such heterogeneity is manifested in cells' response to nutrient availability signals. For example, it is unknown precisely how individual cells respond to glucose removal and whether/how their own experiences may affect their daughter cells' fate choice.

Here, we use a set of live imaging and long-term cell tracking tools, including those designed in this study, to investigate cell fate choice under nutrient depletion conditions at the single cell level. We show that unlike amino acids or glutamine, whose removal immediately halts cell cycle progression, glucose withdrawal does not prevent cells from completing their current cycle. Our results reveal that cells first experiencing glucose withdrawal in S phase give rise to daughter cells that make an equal choice in proliferation or quiescence. In contrast, cells first enduring such condition in G1 phase give rise to daughter cells that predominantly enter quiescence. We identify the p53-p21 axis in regulating this fate choice preference and, importantly, present evidence that such an influence on daughter cells can be traced to the level of p21 inherited from their mother cells. Using an auxin-inducible degron system, we show that artificially degrading p21 under glucose depletion conditions permits cells to enter S phase that consequently accumulates elevated DNA damage. Our results suggest that when facing glucose limitation, mother cells take preemptive steps toward instructing daughter cells to avoid a harmful fate choice of entering S phase.

## Results

### Glucose removal affects cell cycle progression and daughter cells’ ability to proliferate

**Figure 1:**
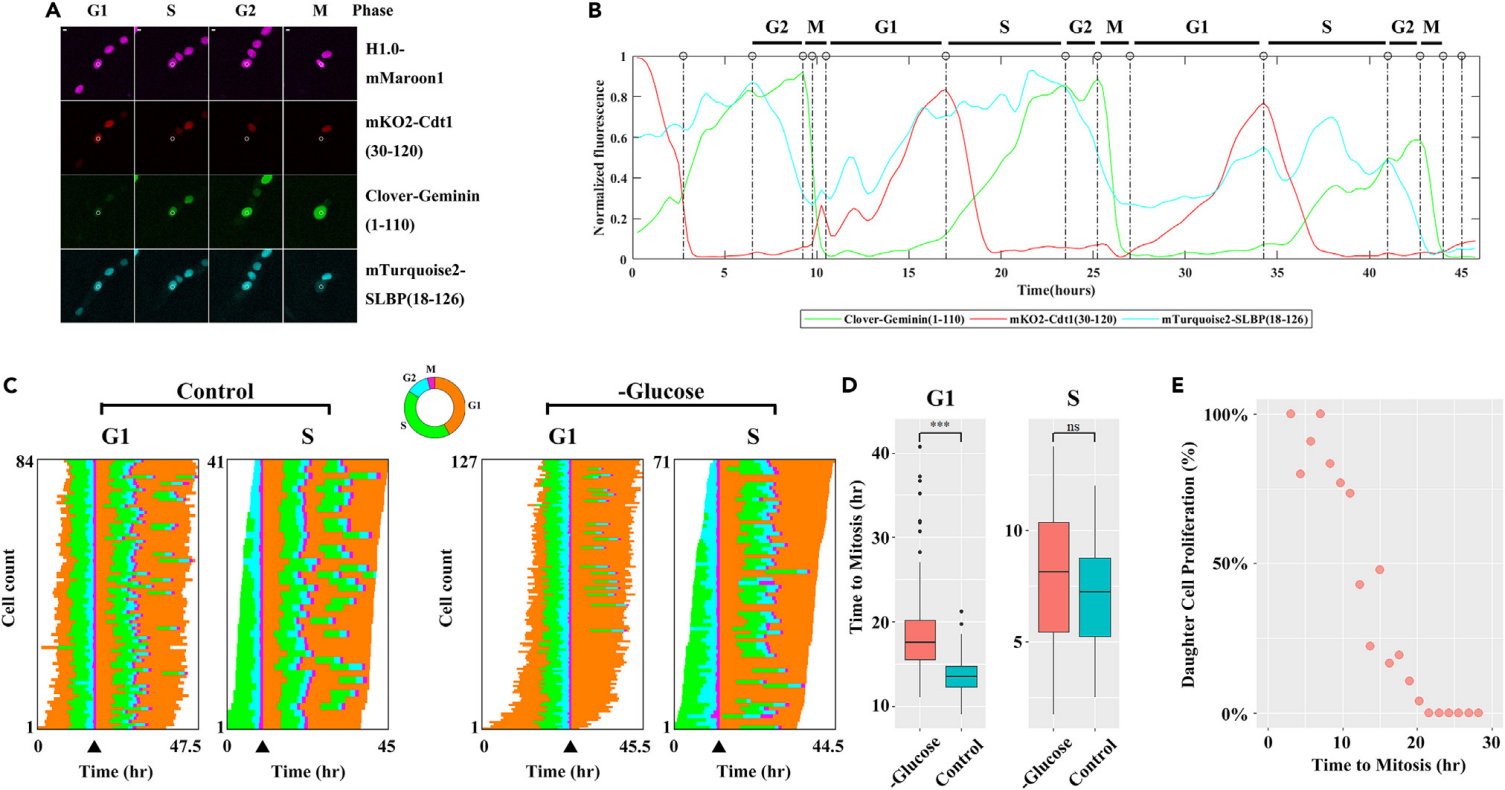
Response of individual cells to glucose removal (A) Shown are representative images of hTERT-RPE1 cells expressing the Fucci4 reporters. This reporter system includes a set of four orthogonal fluorescence: residues 30–120 of Cdt1 fused to mKO2, residues 1–110 of Geminin fused to Clover, residues 18–126 of SLBP fused to mTurquoise2 and Histone1.0 fused to mMarron1. White circles represent the central region of nucleus, which were used for quantifying fluorescent intensities of the corresponding cell. Scale bars: 10 μm. (B) Shown are Fucci4 reporter intensity profiles of a representative cell cultured under normal conditions. Bars at the top label the phases of cell cycle progression according to these profiles. Here, we semi-automatically tracked and segmented individual cells using algorithms developed by TrackMate^18^^,^^19^ and defined cell phases according to the detected fluorescence intensities. As reported previously,^20^^,^^21^ G1 phase had high or increasing levels of Cdt1 reporter activity together with a low Geminin reporter activity. S phase had low or decreasing levels of Cdt1 reporter activity in combination with high or increasing levels of Geminin reporter activity before the peak of SLBP reporter activity. G2 phase was between the peak fluorescence intensity of SLBP and Geminin. M phase was defined by a dramatic change in the histone reporter pattern resulting from chromatin condensation, alignment, and separation during M phase (see also Video S1). The estimation of G1 phase duration in actively proliferating cells in our experiments represents an underestimate because of the inclusion of cells with “truncated” G1 phase in live imaging data. (C) Shown are heatmaps depicting cell cycle progression of individual cells under control or glucose limitation conditions. Cells were grouped according to each cell’s stage at the time of treatment (labeled as G1 and S, representing the two major fractions of captured cells suitable for reliable quantitative analysis). Within each group, cells were ranked by the measured length of G1 or S phase, respectively. All temporal profiles within a group were then aligned by anchoring to each cell’s time of the completion of the first M phase (black arrow). (D) Boxplot showing the time (hr) to the first mitosis for the indicated cells under control (blue) or glucose limitation (red) conditions. ∗∗∗ and “ns” denote p values (Student’s *t* tests) of <10^−3^ and >0.05, respectively. (E) Scatterplot showing the percentage of proliferating daughter cells versus the time it took their corresponding mother cells to reach mitosis under glucose limitation (red) conditions. Here, datapoints shown represented 1.3h bins.

To investigate how the input of nutrient availability feeds into cell cycle control and propagates through cell division, we performed single-cell live imaging studies. Here, we used the untransformed, immortalized retinal pigment epithelial (hTERT-RPE1) cells known to have an intact cell cycle control machinery. We employed a set of different living-imaging reporter systems in our study, including both previously reported and our newly designed ones (see below). As an initial step toward monitoring cell cycle progression in response to nutrient removal, we generated hTERT-RPE1 cells stably expressing the previously described reporters of the Fluorescence Ubiquitin Cell Cycle Indicator 4 (Fucci4) system (see STAR Methods and Figure 1 legend for details).^22^ Our established cells behaved as expected (Figure 1A and Video S1), documenting suitability for time-lapse imaging and long-term cell tracking. For each tracked cell in our study, we quantified reporter intensity profiles as a function of time to infer its G1, S, G2 and M phases (Figure 1B; see also STAR Methods). Actively proliferating cells in our experiments had a duration time of 4.92 ± 2.09 h (mean ± standard deviation), 5.59 ± 1.52 h, 2.19 ± 0.93 h and 0.81 ± 0.13 h for G1, S, G2 and M phases, respectively (see Figure 1B legend for further details).

Video S1. Live imaging of hTERT-RPE1 cells with Fucci4 system, related to Figure 1 Images were taken every 15 min for 30 h, and the playback speed is 10 frames/s. Examples #1 and #2 represented two types of G1 phase arrest. Examples #3 and #4 represented two types of S phase arrest. Example #5 represented a normal cell cycle. Red: Cdt1(30–120); green: Geminin(1–110); cyan: SLBP(18–126); purple: H1.0.

To evaluate the behavior of cell cycle progression with minimal perturbations except nutrient removal, we used actively proliferating, unsynchronized cells; we also serum starvation-synchronized cells in complementary experiments (see below and Supplementary Information). In our analysis, cell viability was unaffected within 48 h after the removal of amino acids, glutamine or glucose separately, but removal of glucose and glutamine together led to a manifest level of cell death (Figure S1). In actively proliferating cells, individual cells begin to experience nutrient removal at their own cell cycle stage at the time of treatment. To facilitate our data analysis, we grouped cells at G1 or S at the time of treatment, the two major fractions of the tracked cells (Figures 1C and S2C). On removal of amino acids or glutamine, most cells (72.5% and 85.3%, respectively) became immediately halted irrespective of their cell cycle stage (Figure S2B), suggesting a strong dependence of cell cycle progression on these nutrients.^23^^,^^24^

In our glucose withdrawal experiments, all the captured cells successfully divided irrespective of their cell cycle stage at the time of treatment (Figure S2B). The duration times under these conditions were 7.39 ± 4.38 h, 6.23 ± 2.05 h, 3.74 ± 1.42 h and 0.84 ± 0.23 h for G1, S, G2 and M (Figure S2A), respectively, revealing a significantly elongated G1 (Student’s *t* test p value = 10^−4^; Figure S2A). It took longer for cells to reach mitosis when glucose removal took place at G1 phase, but not at S phase (Student’s *t* test p values = 10^−4^ and = 0.074, respectively; Figure 1D). In addition, the ability of daughter cells to proliferate was inversely related to the total time duration—including an elongated G1—of mother cells enduring glucose depletion conditions (Pearson correlation *r* = 0.95 and p value = 10^−4^; Figure 1E). These results suggested a sensitivity of G1 phase to glucose depletion in terms of an elongated duration and daughter cell’s ability to proliferate (see also Figure S3 for results in synchronized cells documenting an elongated G0/G1 phase in the absence of glucose).

### Glucose limitation causes daughter cells to have mixed fates of proliferation and quiescence

**Figure 2:**
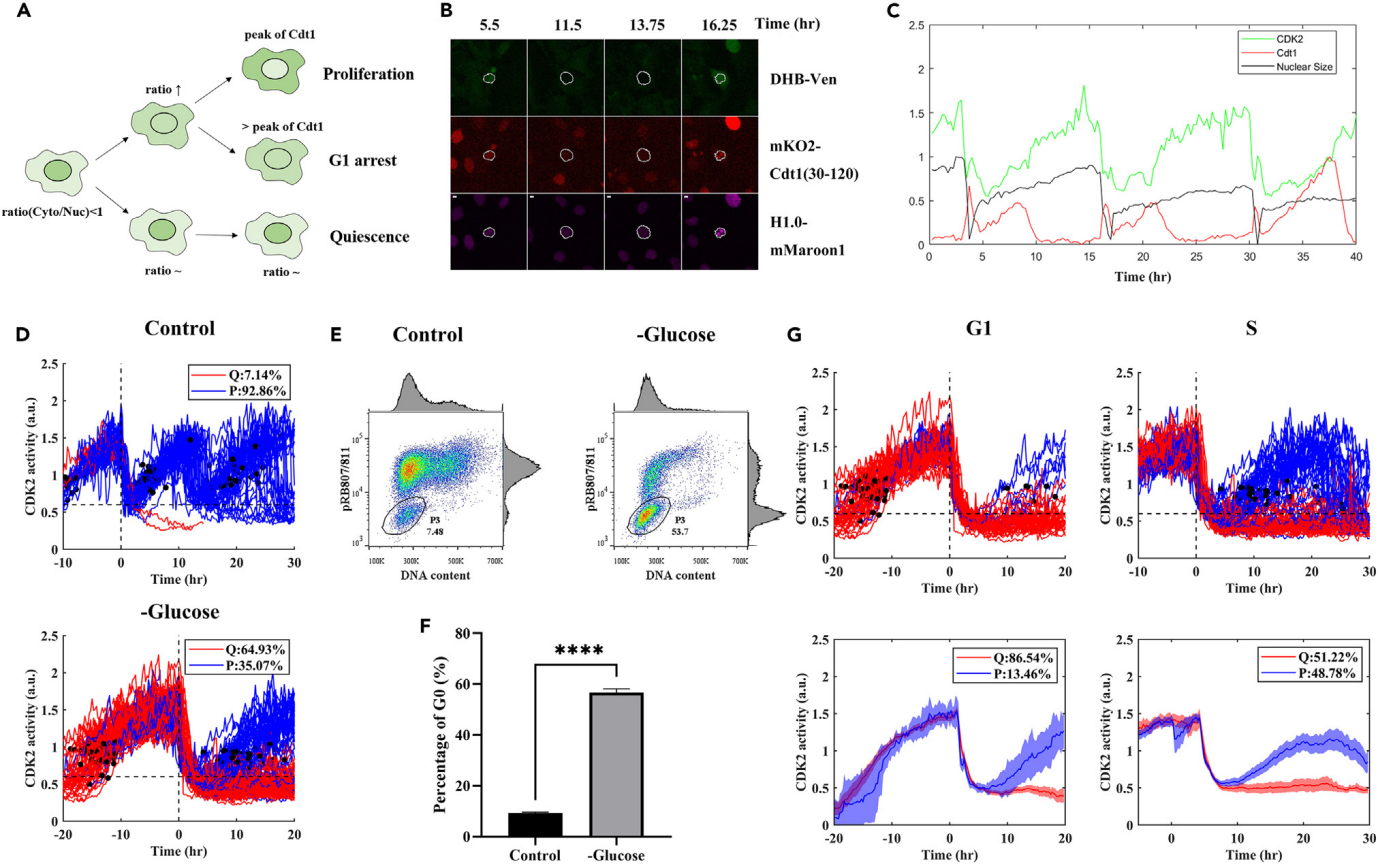
Glucose limitation causes daughter cells to enter quiescence (A) Schematic diagram showing the expected behavior of cells at the indicated states. Here, we used a CDK2 activity sensor to detect fluorescent signals of DHB-mVenus,^25^ and the cytoplasmic to nuclear ratio (Cyt/Nuc) as a readout. Quiescent cells were identified as those whose Cyt/Nuc ratio did not increase, G1 arrested cells were identified as those whose Cyt/Nuc ratio increased but Cdt1 activity was below its peak, and proliferative cells were identified as those whose Cyt/Nuc ratio increased and Cdt1 activity reached its peak. (B) Representative images of hTERT-RPE1 cells stably expressing DHB-mVenus, mKO2-Cdt and H1.0-mMaroon1 cultured under normal conditions (see also Video S2). White circles demarcate the nuclear regions, which were used for measuring nuclear size and nuclear signals. Scale bars: 10 μm. (C) Shown are intensity profiles of a representative cell cultured under normal conditions. Green: CDK2 activity (Cyt/Nuc of DHB-mVenus); red: Cdt1 intensity (normalized to maximum); black: nuclear size (normalized to maximum). (D) Shown are dynamic profiles of CDK2 activity in cells under control (*N* = 42; upper) and glucose depletion (*N* = 265; lower) conditions. Individual profiles were aligned by anchoring to the 1^st^ division time. Red: quiescence (7.14% in upper and 64.93% in lower); blue: proliferation (92.86% in upper and 35.07% in lower). Black dots mark the time at which a tracked cell reached its peak Cdt1 intensity. (E) Shown are data of flow cytometry analysis of cells cultured under control and glucose depletion conditions for 48 h. The pRB807/811 antibody and propidium iodide (to measure DNA content) were used for staining. Quiescent cells were identified as low pRB807/811 and low DNA content, marked as the P3 gate. (F) Shown percentages of quiescent cells measured from P3 in (E). Error bars represent standard errors of the mean (SEM). Student’s *t* test p value < 10^−3^. (G) Shown are CDK2 activity profiles in cells grouped according to their stage (G1 or S) at the time of treatment (top panels). Blue and red colors denote cells that followed a proliferative or quiescent path, respectively. Lower panels show the corresponding mean profiles with 95% confidence interval.

Glucose starvation has been reported to lead to G1 arrest resulting from a decrease in G1 phase cyclins.^24^ Because mother cells could successfully complete their current cell cycle upon glucose removal (Figure S2B), we set out to monitor daughter cell behavior, particularly with regard to the status of G1 arrest or quiescence of daughter cells. Here, we established hTERT-RPE1 cells stably expressing a CDK2 activity sensor with Cdt1 and Histone1.0 reporters (Figures 2B and 2C). This sensor contains the fluorescent protein mVenus fused to residues 994–1087 of human DNA helicase B (DHB) consisting of four CDK2 consensus phosphorylation sites on serine, a nuclear localization signal, and a nuclear export signal.^25^ The cytoplasmic to nuclear ratio (Cyt/Nuc) of the detected fluorescent signals of this sensor provides a readout of CDK2 activity (Figure 2A). Previous biochemical studies using synchronized cells through serum starvation and replenishment documented an increase in CDK2 activity upon entering G1 and peaking at M phase.^8^ We performed single cell tracking to verify that our reporter system behaved as expected.^8^^,^^25^ Specifically, cells were first arrested (with 24 h serum starvation) and then re-stimulated (with serum) to enter cell cycle. Our results showed that on serum replenishment, low CDK2 activity persisted for an additional ∼10 h before it began to slowly and monotonically ascend, reaching a peak at mitosis marked by nuclear envelope breakdown (Figure S4A and Video S2).

Video S2. Live Imaging of hTERT-RPE1 cells with a CDK2 sensor under normal conditions, related to Figure 2 Images were taken every 15 min for 30 h, and the playback speed is 10 frames/s. Red: Cdt1(30–120); green: DHB; purple: H1.0.

To evaluate daughter cell fate, we monitored CDK2 activity in individual cells over a period of 48 h without glucose (Figure 2D). CDK2 activity in daughter cells derived from mother cells undergoing persistent glucose depletion exhibited an overall bifurcated distribution. Over half of the daughter cells had a low CDK2 activity (Cyt/Nuc ratio ≈0.6), suggesting that these daughter cells entered quiescence rather than G1 arrest. We also used DNA content (PI) and the activity of a proliferation marker, phospho-Rb (Ser807/811), to verify cell cycle status (Figures 2E and 2F), confirming an increased fraction of cells with low PI and phospho-Rb (Ser807/811) signals in the absence of glucose. Importantly, when daughter cells were evaluated as separate groups according to their mother cell’s stage at the time of treatment, the percentage of quiescence in the G1 group was higher than in the S group (86.54% and 51.22% respectively) (Figure 2G). These results, together with those obtained with the Fucci4 system described above (Figure 1E), suggested that the proliferation-quiescence fate choice of daughter cells was associated with their mother cell’s stage at the time of glucose removal. We note that as shown in Figure S4, whereas both serum removal and glucose withdrawal could affect daughter cell fate choice, their response characteristics were different, suggestive of distinct regulatory mechanisms involved (see below for additional molecular evidence). In particular, although cells responded to a broad range of serum concentrations in a dose-dependent manner^15^ (Figure S4B), cells did not respond to glucose limitation until its medium concentration reached 0.1 mM or lower (Figures S4C and S4D; see also Figure S3 for an identical responding range to glucose limitation for mother cells’ G1 phase elongation).

### Glucose withdrawal-induced fate choice preference is mediated by p21

**Figure 3:**
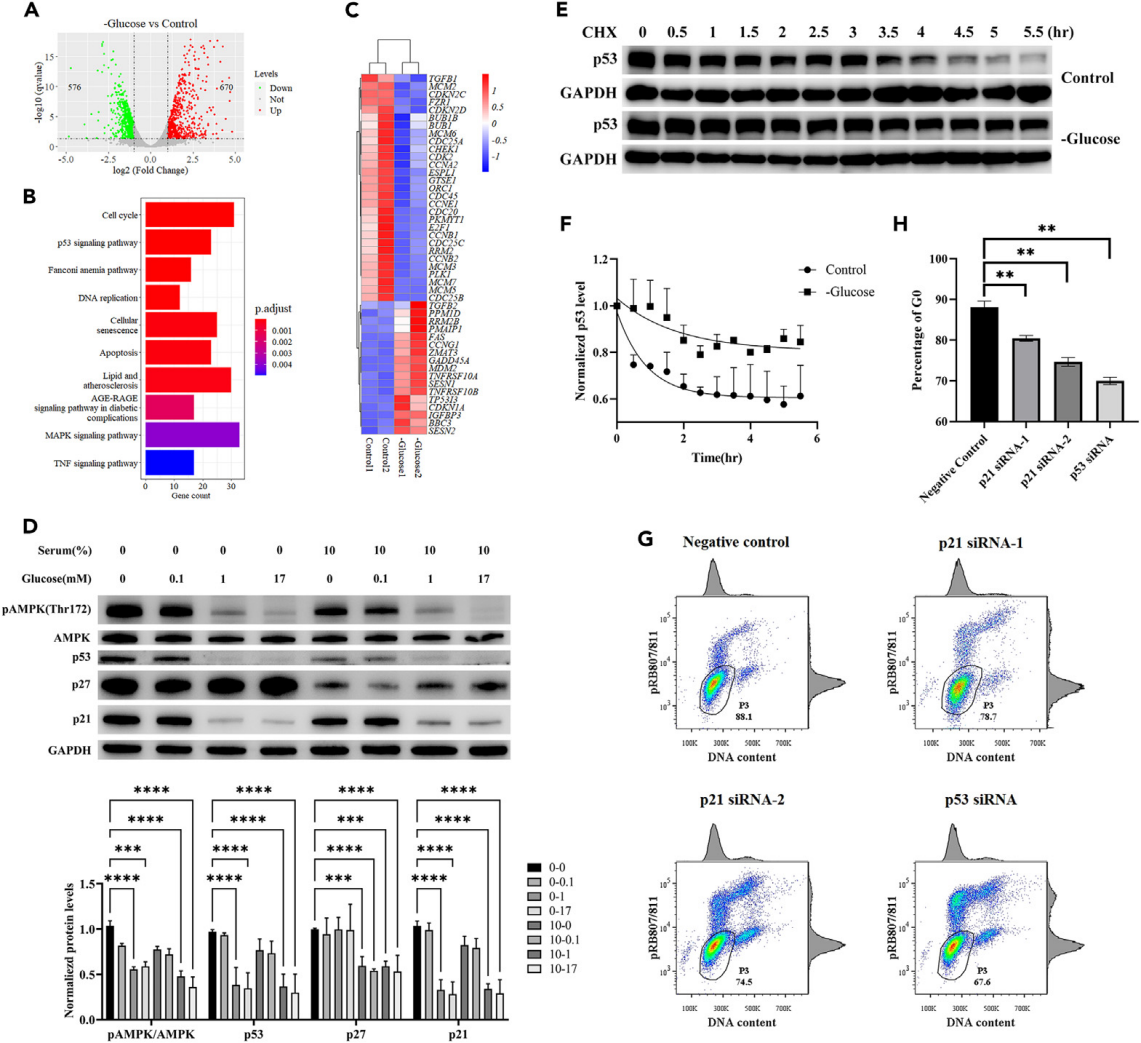
The p53-p21 regulatory axis contributes to proliferation-quiescence fate choice (A) Shown is a volcano plot of RNA-seq data exhibiting fold change (log 2) between cells cultured without glucose for 24 h and control cells. Horizontal dashed line marks adjusted p value = 0.05 and vertical dashed lines mark log2(fold change) = ±1 thresholds for differentially expressed genes. (B) Shown are top 10 pathways that were enriched (with lowest adjusted p values) with differentially expressed genes. (C) Shown is a heatmap (log2 fold change) of the differentially expressed genes that belong to the functional categories of cell cycle and p53 signaling pathway. (D) Shown Western blot results detecting AMPK phosphorylation at Thr172, total AMPK, p53, p27, p21 and GAPDH in cells that had been cultured for 24 h in media containing the indicated concentrations of glucose and serum. Quantification was performed by normalization to the corresponding GAPDH levels. Error bars represent SEMs of three replicate experiments. ∗∗∗ and ∗∗∗∗ represent p values of Dunnett’s multiple comparisons test <10^−3^ or <10^−4^, respectively. One-way ANOVA p values against glucose concentration = 0, 0.1, 1 and 17 mM for pAMPK/AMPK, p53, p27 and p21, respectively; ANOVA p values against serum concentration = 0 and 10%. (E) Western blot analysis detecting p53 and GAPDH at the indicated time after addition of 2.5 μg/mL cycloheximide (CHX) to hTERT-RPE1 cells cultured in media containing 0 or 17mM glucose. (F) Quantification of results shown in (E) with p53 normalized to GAPDH. Error bars represent SEMs of three replicate experiments. (G) Shown are results of flow cytometry analysis performed similarly to that in Figure 2E, except that hTERT-RPE1 cells had been transfected with a negative control siRNA, two p21 siRNAs and a p53 siRNA for 24 h, followed by glucose depletion for 24 h. (H) Shown are the percentages of quiescent cells measured from the P3 gate in (G). Error bars represent SEMs of three replicate experiments. ∗∗ represents p values of Dunnett’s multiple comparisons test <0.01. One-way ANOVA p values against siRNA of negative control, p21 siRNA-1, p21 siRNA-2 and p53 siRNA for percentages of quiescent cells.

To gain insights into molecular responses to glucose removal, we performed an RNA-seq analysis (Figure 3A). Cell cycle, p53 signaling pathway and DNA replication were among the top 10 pathways that were enriched in differentially expressed genes in the absence of glucose (Figure 3B). Specifically, genes encoding mini-chromosome maintenance complex proteins (all of which except Mcm4) were significantly downregulated, suggesting an inhibitory effect of glucose limitation on DNA biosynthesis, an energy-costly process (Figure 3C). In addition, p53 target genes, including *CDKN1A* (encoding p21), *TP53I3* and *MDM2*, were significantly upregulated (Figure 3C).

To evaluate the p53 signaling response, we measured protein levels of p53, p27 and p21 under conditions of varying concentrations of glucose and serum (Figure 3D). Both p27 and p21 are known to compete with cyclin proteins for CDK2 and promote quiescence.^26^ In our experiments, whereas the increase in p27 protein level was associated with serum depletion, protein level increase for both p53 and p21 was associated with glucose removal. Because *p53* mRNA level was not significantly changed in our RNA-seq data (adjusted p value = 0.073; fold change = 1.78), we measured p53 protein half-life and found it to be increased from 0.61 to 1.26 h under glucose deprivation, suggestive of regulation of p53 protein stability (Figures 3E and 3F). In our experiments, we also confirmed AMPK activation as monitored by its phosphorylation at threonine 172, pAMPK(Thr172), under glucose depletion conditions as expected.^27^ Importantly, both AMPK activation and p53-p21 activation took place when glucose concentration was 0.1 mM or lower (Figure 3D), indicating an identical responding range to glucose limitation for both molecular signaling and cell cycle progression (see also Figure S4). Finally, flow cytometry analysis showed that siRNAs against *p53* or *p21* reduced the fraction of quiescent cells without glucose (Figures 3G and 3H; see Figure S5 for knockdown efficiencies). Together, these results suggested a contribution of both of these activities to regulating the proliferation-quiescence fate choice under glucose limitation conditions.

### p21-mediated daughter cell fate choice preference originates from mother cells

To further investigate the contribution of the p53-p21 regulatory axis to cell fate decisions under glucose depletion conditions, we devised a system for monitoring the dynamics of p21 through live imaging. Here, we generated hTERT-RPE1 cells stably expressing the CDK2 activity sensor and the Histone H1.0 reporter, along with a newly engineered p21 reporter. This p21 reporter was a fusion protein with a C-terminal tag of miniIAA7-mTurquoise2 suitable for both live imaging and, as explained below, for induced p21 protein degradation. Through CRISPR/Cas9-mediated homologous recombination, we introduced this reporter gene at both alleles of the endogenous *CDKN1A* gene (Figures S6A and S6B). Our p21-miniIAA7-mTurquoise2 fusion protein behaved similarly to the native p21 protein in that it was localized exclusively to the nucleus, induced upon glucose removal (Figure S6C), and functionally active in interacting with CDK2 (Figure S6D).

**Figure 4:**
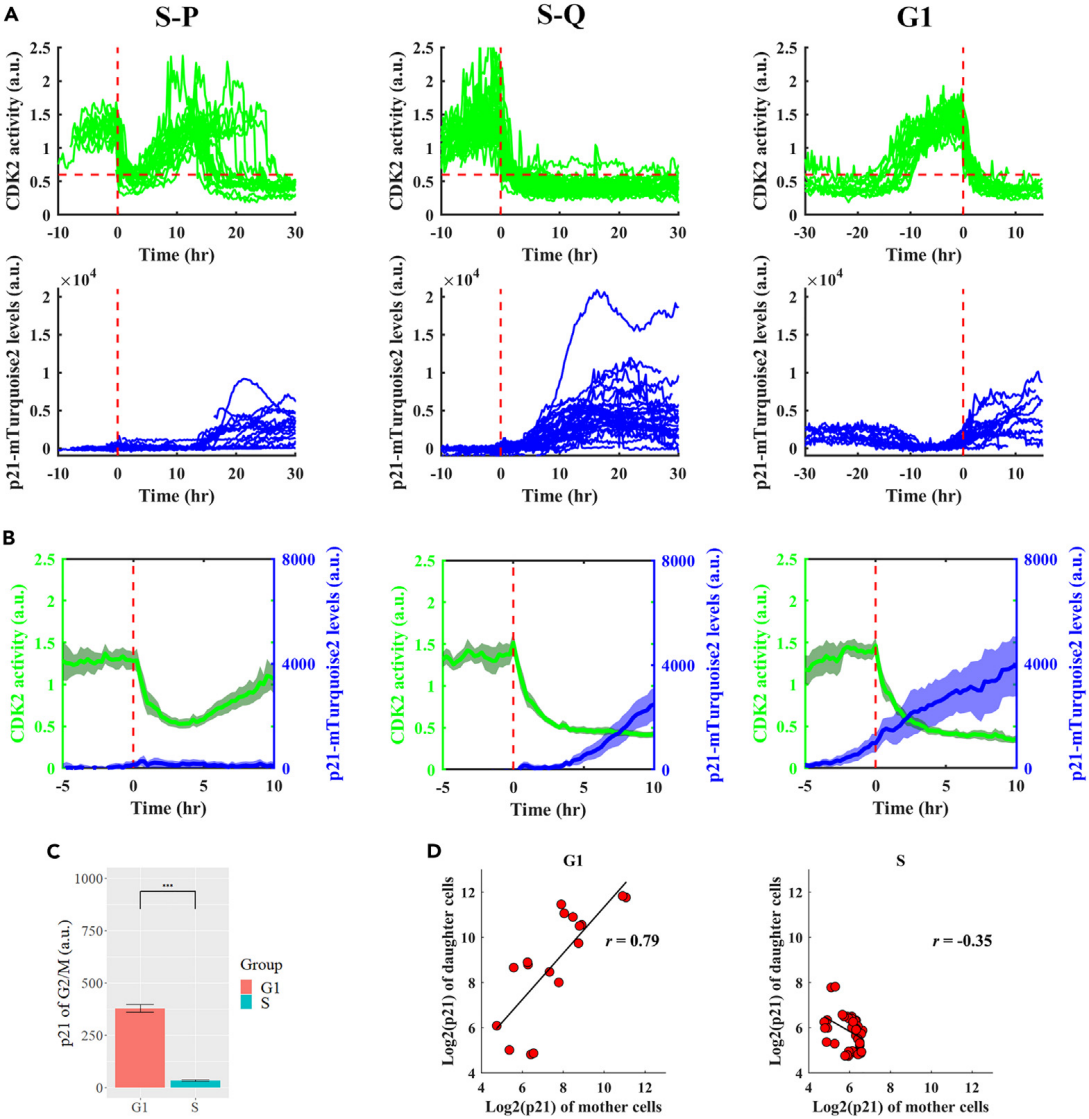
Dynamic expression of p21 in cells under glucose deprivation conditions (A) Shown is profiles of CDK2 activity (green) and p21-mimiIAA7-mTurquoise2 intensity (blue) of a representative cell cultured under glucose depletion conditions. Three groups of cells were analyzed separately (see text for details): S-P (*N* = 18), S-Q (*N* = 34), and G1 (*N* = 18). In each panel, individual profiles were aligned by anchoring each cell to the 1^st^ mitosis (Time = 0). Horizontal dashed lines represent CDK2 activity (Cyt/Nuc ratio) = 0.6. (B) Mean profiles of CDK2 activity (green) and p21 reporter intensity (blue) for the indicated groups. The shaded bands represent 95% confidence intervals. The time window shown spans the last 5 h of mother cells and the first 10 h of daughter cells. (C) Boxplot of p21 reporter intensity in G2/M phase between G1 group (red) and S group (blue). ∗∗∗ represent p values of Student’s *t* tests <10^−3^. (D) Scatterplots of p21 reporter intensities measured from mother-daughter cell pairs. Intensities were measured as the mean values within 5 h windows before (i.e. mother cells) and after mitosis (daughter cells). The mother-daughter cell pairs were grouped according to the cell cycle phase (G1 or S) of the mother cell at the time of glucose removal. *N* = 18 and 73 for G1 and S groups, respectively; Pearson correlation *R* = 0.79 and −0.35; p value = 10^−3^ and = 0.002, respectively.

We performed long-term tracking of cells containing the fusion reporter to follow p21 expression dynamics in response to glucose removal. According to mother cell’s stage at the time of glucose removal and daughter cell fate choice, we divided the tracked cells into three groups for further analysis: mother cells at S phase (at the time of treatment) with proliferating daughter cells (S-P), mother cells at S phase with quiescent daughter cells (S-Q) and mother cells at G1 phase which gave rise to quiescent daughter cells (G1) (Figure 4A). For cells within each group, we aligned the temporal profiles to each cell’s first mitotic time. We found that the distribution of p21 level in daughter cells differed among the three groups (Figure 4A). To better evaluate the onset of p21 accumulation, we zoomed into a 10 h-time window spanning 5 h before and after mitosis (Figure 4B). Our results showed that for cells in the G1 group, the onset of a robust accumulation of p21 protein began before mitotic division although a modest elevation could be detected as early as G1 phase (see Figure 4A for the time window between −30 and −20 h for the G1 group; see also discussion for implications of this finding). This is in contrast to cells in S-Q and S-P groups where p21 accumulation began only after mitosis (Figure 4C). For cells in the G1 group, a daughter cell’s p21 level in early G0/G1 phase was positively correlated with its mother cell’s p21 level at G2/M phase (Figure 4D). These results document that depending on mother cell’s nutrient experience, p21 accumulation could begin in mother cells to be suitable to exert its influence on daughter cell fate choice.

### Daughter cells inherit p21 from mother cells enduring glucose removal starting in G1

**Figure 5:**
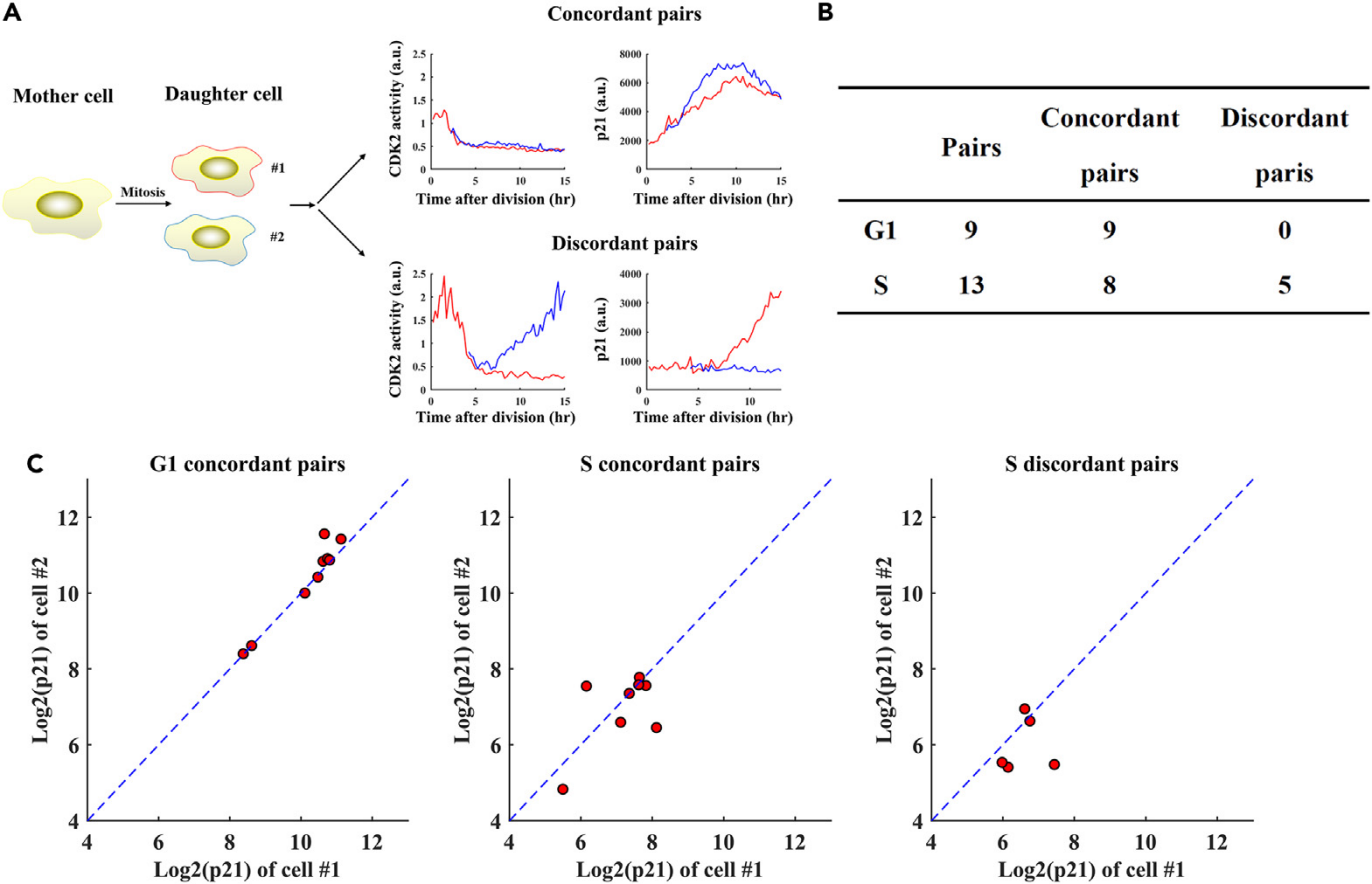
Daughter cells inheriting high levels of p21 enter quiescence (A) Schematic diagram of cell division with two daughter cells making either same or divergent fate choices, denoted as concordant or discordant pairs, respectively. Also shown are p21 reporter intensity and CDK2 activity profiles in a representative concordant pair and a representative discordant pair of daughter cells. CDK2 activity was used to define quiescence-proliferation cell fate as described in Figure 2. (B) Detected concordant and discordant pairs of daughter cells that were derived from mother cells that began to experience glucose deprivation at either G1 or S phase. (C) Representative scatterplots of p21 reporter intensities measured from the indicated pairs. p21 reporter intensities shown represent the mean over a 5 h window. *N* = 9, 8 and 5 for G1 concordant pairs, S concordant pairs, and S discordant pairs, respectively. See text for additional information about statistical evaluations of the intensity data in each group.

To test the possibility that a daughter cell’s fate choice is influenced by an inheritance from its mother cell, we tracked sibling daughter cells under sustained glucose depletion. Sibling cells derived from symmetric cell division are expected to inherit equal amounts of key regulatory factors from their mother cell.^10^^,^^28^ If the inhibitor protein p21 began to accumulate in the mother cell to be distributed equally to sibling cells, the two cells would make concordant decisions (with respect to proliferation or quiescence) depending on the inherited p21 level. Otherwise, sibling cells would make independent decisions, i.e., the decisions could be either concordant or discordant (Figure 5A). Among the detected 9 pairs of sibling cells in the G1 group, all made concordant decisions. In contrast, among the detected 13 pairs of sibling cells in the S group, 8 pairs made concordant decisions whereas 5 pairs made discordant decisions (Figure 5B).

It is well documented that p21 activity is sufficient to drive cells toward quiescence.^11^^,^^15^^,^^17^^,^^29^ To determine whether p21 could indeed be an inheritance under our experimental conditions, we directly compared p21 levels in two sibling cells in our tracked pairs (Figure 5C). We used coefficient of determination (*R*^2^) to measure the discrepancy within non-ordered sibling pairs in each of the three groups. The better the sibling cells resembled each other in their p21 level for a given group, the closer to 1 the *R*^2^ value. For the G1 concordant group, S concordant group and S discordant group, *R*^2^ = 0.90, 0.19 and 0.05, respectively. These results showed that sibling cells with the highest resemblance in p21 levels were from mother cells that began experiencing glucose removal at G1 phase. They further supported the hypothesis that a mother cell’s nutrient experience can impact its daughter cells’ fate choice through an inheritance of p21.

### Rapid degradation of p21 overrides glucose depletion-induced quiescence and causes abnormal cell cycle

**Figure 6:**
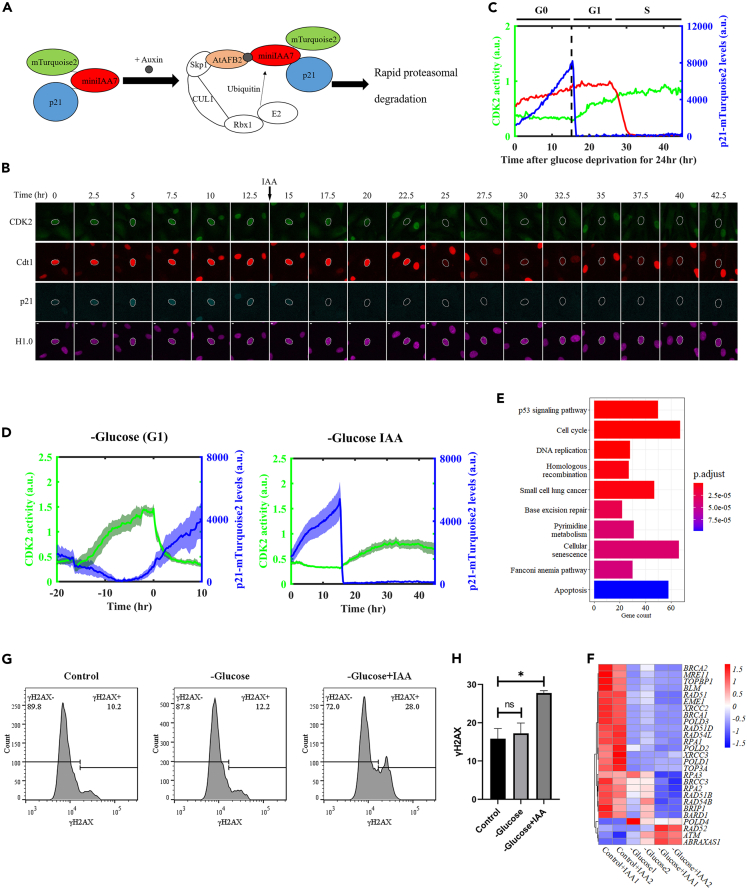
p21-mediated quiescence under glucose deprivation conditions prevents cells from entering an abnormal cell cycle (A) Schematic diagram of rapid p21 degradation using the auxin-inducible degron (AID) system. (B) Time-lapse imaging of a representative cell with the p21 AID system. Here, cells were first cultured in medium without glucose for 24 h and then imaged for 48 h with 500 μM IAA being added at 12.5 h after initiation of imaging. Green: DHB-mVenus (CDK2 activity sensor); red: mKO2-Cdt1; blue: p21-miniIAA7-mTurquoises; pink: H1.0-mMaroon1. Scale bar: 10 μm. (C) Quantitative profiles of a representative cell with the p21 AID system under sustained glucose depletion and with induced p21 degradation as described in panel B. Green: CDK2 activity; red: Cdt1 intensity; blue: p21 intensity. (D) Mean profiles of CDK2 activity (green) and p21 intensity (blue). Right panel: cells with the p21 AID system (*N* = 32) were cultured without glucose for 24 h and then subjected to live imaging analysis, followed by the addition of 500 μM IAA 15 h later. Left panel represents a control without the artificial p21 degradation and is a replotted version of data from Figure 4. Compare the CDK2 activity in these two panels: the peak in the left panel marks a successful completion of cell cycle to reach mitosis (marked at 0 h), whereas the right panel depicts a failure to reach such a peak. The shaded bands represent 95% confidence intervals. (E) Top 10 pathways that were enriched (with lowest adjusted p values) with differentially expressed genes comparing IAA-treated samples under glucose depletion conditions and IAA-treated samples in normal culture. (F) A heatmap showing log2 fold changes of differentially expressed genes that have functions in base excision repair. (G) Flow cytometry analysis detecting γH2AX in cells cultured under normal conditions, glucose depletion, or glucose depletion with IAA treatment. (H) Quantification of γH2AX level from (G). Error bars represent SEMs of three independent experiments. ∗ and “ns” represent p values of Student’s *t* tests <0.05 and >0.05, respectively.

The p21 fusion protein described above contained miniIAA7, a tag for the auxin-inducible degron (AID) system.^30^ This system, which was designed for induced protein degradation, consists of the miniIAA7 tag and a separately expressed F box protein (*At*AFB2) in the same cell (Figure 6A). To verify the efficiency of this degradation system in our experimental setup, we used a scheme as depicted in Figure S7B, where cells were first treated with Nutlin-3a for 24 h to allow p21 protein to accumulate, followed by addition of the inducer Indole-3-acetic acid (IAA). As documented by both live imaging and Western blot analysis, our p21 fusion protein became undetectable within 2 h after IAA addition (Figures 6B and S7B and Video S3).

Video S3. Live Imaging of hTERT-RPE1 cells with the p21 AID system, related to Figure 6 Cells were first cultured in medium with 10 μM Nutlin-3 for 24 h and then imaged for 30 h with 500 μM IAA being added at 4 h after initiation of imaging. Images were taken every 15 min for an additional 7 h, and the playback speed is 10 frames/s. Green: p21; red: AtAFB2.

To evaluate how controlled degradation of p21 affects daughter cell fate choice under glucose depletion conditions, we combined the p21 AID system with the CDK2 activity sensor, the mKO2-Cdt1 reporter and Histone H1.0-mMaroon1 (Figures 6B and 6C). Here, cells that had been cultured without glucose for 39 h accumulating p21 fusion protein to a high level were induced by IAA for its rapid degradation (Figure 6D blue). In our analysis, CDK2 activity did not ascend until p21 became undetectable (Figure 6D green), which led cells to progress successfully over both G0/G1 and G1/S transitions. However, the level of CDK2 activity plateaued in S phase and never reached as high as that of proliferative cells under the same glucose depletion conditions (Figure 2D blue). Indeed, these cells stayed arrested in S phase and never divided within the 48-h monitoring window (see Figure S8 for Cdt1 activities in individual cells).

To characterize the molecular responses to “forced” p21 degradation under glucose depletion conditions, we performed an RNA-seq analysis under our experimental setup. We found that in addition to cell cycle and p53 signaling pathways, DNA repair pathways were also enriched with differentially expressed genes in IAA-treated samples under control or glucose depletion conditions (Figure 6E). In particular, genes involved in base excision repair were downregulated, suggesting a possibility of a compromised DNA repair system that could result in an accumulation of DNA damage upon entering S phase (Figure 6F). To test this idea directly, we performed a flow cytometry analysis of cells stained with the DNA damage marker γH2AX. Our results revealed a significant increase in γH2AX intensity in IAA-treated glucose-depleted cells, confirming an increased level of DNA damage (Figures 6G and 6H). Collectively, these results suggested that p21-mediated quiescence is protective to cells facing glucose limitation.

## Discussion

Our current study was designed to address several specific questions with regard to the impact of nutrient depletion on cell fate choice as outlined in introduction. Our results suggest that glucose depletion-induced quiescence is a deliberate cell fate choice. This choice is made by daughter cells and is subject to influence by their mother cells’ experiences in enduring glucose removal. The effect of glucose withdrawal is distinct from that of the removal of amino acids or glutamine, where cells halt their cell cycle progression without being able to complete the current cycle. This distinction argues against the idea that glucose depletion-induced quiescence is purely a passive consequence of cell’s incapability to proceed in cell cycle because of, e.g., energy exhaustion. It was reported previously that in synchronized HeLa cells, glucose is needed for G1-S transition but not for progression through S phase, whereas glutamine plays a critical role in S phase progression.^23^ It remains to be determined how individual cells sense and integrate the availability of various nutrients at the level of metabolism and energy production in making their fate choice. Importantly, our results suggest that daughter cell’s quiescence under glucose depletion conditions is mediated by molecular signaling and is of deliberate and protective nature.

**Figure 7:**
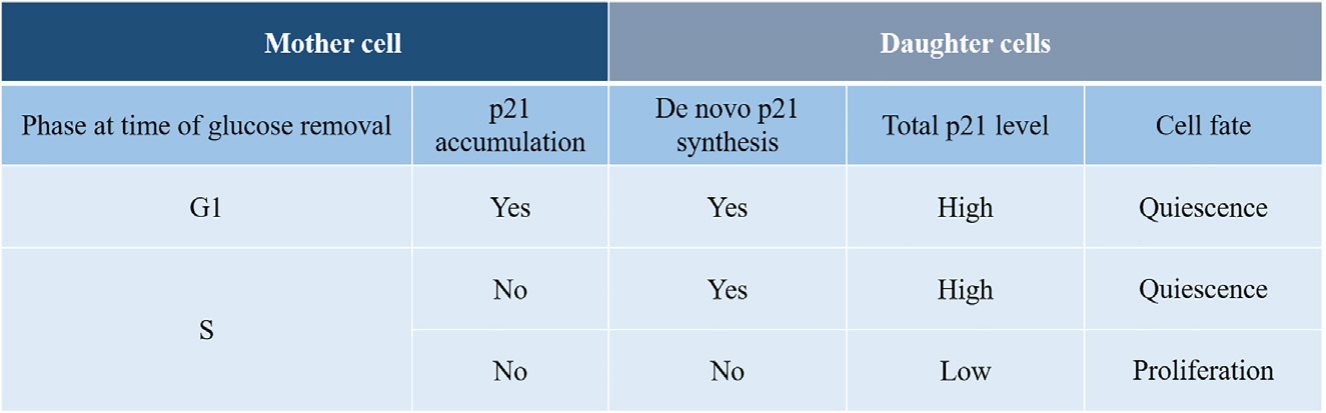
Model of p21 determinants in cell fate under glucose limitation

Our results show that for daughter cells derived from mother cells that endure glucose removal starting in G1, their quiescence is influenced by p21 as an inheritance from mother cells. These mother cells begin a meaningful accumulation of p21 protein during G2/M phase. Consequently, their daughter cells inheriting heightened levels of p21 are driven toward adopting a quiescence fate choice. For these daughter cells, the inherited p21 level represents a deterministic component in their cell fate decision. This is distinct from daughter cells derived from mother cells that begin to endure glucose depletion in S. In this latter case, *de novo* p21 accumulation takes place only in daughter cells with little inheritance, thus representing a stochastic component in the proliferation-quiescence decision (Figure 7). These results suggest that the cell cycle checkpoint through which glucose limitation signals are funneled may be placed at either R1 or R2 depending on the mother cells’ nutrient depletion experiences. Our results show that G1, but not S, is lengthened by ∼2 h in mother cells without glucose, a finding that is further confirmed using cells that are synchronized by serum depletion and replenishment (Figure S3). Perhaps this elongated G1 provides a special, adaptive period for mother cells to prepare for p21 accumulation, a period missing in cells that begin enduring glucose removal in S. The concept that daughter cells can inherit p21 from mother cells has been described in previous studies^11^^,^^13^^,^^14^ and, therefore, the significance of our work is not about confirming or advancing this concept in a generic way. Rather, our study documents specifically an influence of this inheritance on daughter cell fate choice under glucose depletion conditions and, importantly, the protective nature of this inheritance in guiding daughter cells away from a fate choice with harmful consequences.

Our results show that induced p21 degradation under glucose depletion conditions allows cells to overcome quiescence and enter S phase. But this is a failed S phase that is marked by both a subsequent S phase arrest and accumulation of an elevated level of DNA damage. Thus, although DNA damage is known to cause p21 induction and prevent cell proliferation,^11^ glucose depletion-induced p21 prevents cells from accumulating DNA damage through avoidance of an otherwise harmful proliferation choice. Our findings thus further enrich an emerging picture where p21 plays a key role in stress response and cell cycle control.^11^^,^^13^^,^^25^^,^^29^^,^^31^ It remains to be understood precisely how daughter cells that are “tricked” into entering S phase in the absence of glucose accumulate DNA damage in relation to DNA replication, e.g., whether DNA replication may become error prone under glucose depletion conditions or DNA repair may become ineffective without a sufficient energy source. In our experiments, DNA repair genes are down-regulated upon glucose removal and such dysregulation is further exacerbated by forced p21 degradation (Figure 6G), suggesting a contribution of molecular signaling and transcription control to the observed elevation of DNA damage levels.

A specific contribution of our work is the design of the p21 fusion protein suitable for both long-term cell tracking and auxin-induced p21 degradation. This has enabled us to uncover not only the dynamics of p21 protein accumulation in mother cells facing glucose limitation and the inheritance in daughter cells, but also the preemptive nature of the steps taken by mother cells in guiding daughter cells away from a harmful choice. Our system to manipulate p21 level is based on a design that could be viewed somewhat as an opposite or mirror image of a previously described method.^25^ In particular, although our system was designed to rapidly degrade glucose depletion-induced p21 thus documenting the consequence of removing p21 under such conditions, the earlier method used the TMP system to induce p21 expression under normal conditions thus documenting the sufficiency of p21 activity in inducing quiescence. Our system will be useful in evaluating the dynamics p21 protein accumulation under other conditions and, importantly, the biological consequences of its induced removal. Our study thus points to a general value of complementary or independent approaches in deepening our understanding of the biology of p21 in cell cycle control.

Glucose is a key carbon source for generating ATP in mammalian cells.^32^^,^^33^ Its acute removal from the medium leads to changes in both metabolism and cell proliferation,^34^^,^^35^ and such changes are likely mediated by AMPK activation.^6^^,^^27^^,^^36^^,^^37^ Our results identify the p53-p21 axis in regulating fate choice under glucose depletion conditions with an AMPK activation and, importantly, we document an identical responding range to glucose limitation for both molecular and cellular responses. This pathway is also operative in an untransformed cell line, HL-7702, and treatment with the AMPK activator A-769662 is sufficient to cause an increase in p53 and p21 protein levels and bifurcation in daughter cell fate choice under normal glucose conditions (our unpublished results), further supporting the role of AMPK activation in driving cell fate toward quiescence. In our experiments described in this study, serum starvation and glucose depletion elicit distinct molecular responses that lead to quiescence, through the accumulation of p27 and p21 proteins, respectively. Cells also exhibit distinct response characteristics with regard to both the status of dose dependence and the range of responding concentrations. Our finding that “forced” degradation of p21 under glucose depletion conditions is sufficient to permit cells to enter S phase further supports the specificity of the molecular responses dissected by our experiments. It has been noted that although p53 activation induced by DNA damage is associated with reduced cell survival, p53 activation induced by glucose deprivation may be beneficial to cell survival.^6^ Our finding that p53-activated p21 expression under glucose depletion conditions can preemptively guide daughter cells away from DNA damage-prone S phase and cell death (data not shown) may help explain this apparently paradoxical role of p53. We note that although induced p21 degradation can “trick” cells into entering cell cycle in the absence of glucose, cell cycle progression is comprised with CDK2 activity plateauing in an arrested S phase. The precise mechanism underlying these defects at both molecular and cellular levels remains to be understood and our results suggest that activation of the p53-p21 axis is unlikely the only change taking place in cells facing glucose limitation. Our strategy of “tricking” cells into entering cell cycle when facing glucose limitation represents a useful tool in future investigations.

Both AMPK activation and DNA damage can induce p21 accumulation.^6^^,^^14^ As documented previously,^11^ p21 accumulation is induced by DNA damage that takes place primarily during S phase. Importantly, AMPK activation by A-769662 does not induce DNA damage as evaluated by a DNA damage reporter system (our unpublished results). In our analysis (Figure 4A, G1 group), mother cells that begin to experience glucose withdrawal at G1 already exhibit a modest elevation of p21 level before S phase (see time window between −30 and −20 h). This modest p21 accumulation is evidently insufficient to reach a threshold to affect cell cycle progression because all of these mother cells can successfully complete their current cycle (Figure S2B). As these mother cells reach S phase, p21 level becomes suppressed, followed by a more robust and sustained elevation at G2/M and beyond. These results suggest that under glucose depletion conditions in our analysis, cells can accumulate p21 in a manner that is independent of DNA damage incurred during S phase. They also suggest that although the meaningful accumulation of p21 begins to takes place at G2/M, activation of the pathway is initiated in G1 phase of these mother cells. It remains to be investigated precisely how individual mother cells facing glucose limitation regulate the relative dynamics of p21 transcript accumulation and p21 protein accumulation in instructing daughter cell fate choice.

### Limitations of the study

Our study takes a systems approach toward understanding how individual cells’ experiences in enduing nutrient depletion impact cell fate choice for themselves and for their daughter cells. It uncovers a role of p21 accumulation in mother cells in driving daughter cells toward quiescence. Our results indicate that glucose depletion-induced AMPK signaling pathway is sufficient to induce p21, and this signaling pathway is operative in two independent cell lines tested. However, a combined treatment of glucose withdrawal and AMPK inhibitor had a deleterious effect on cell survival (our unpublished results), preventing a direct test of whether this signaling pathway is solely responsible for p21 accumulation in response to glucose depletion in our analysis. Nevertheless, our existing evidence is in favor of the hypothesis that DNA damage and AMPK activation are two distinct, and likely unrelated, triggers for p21 accumulation. Collectively, our results suggest that glucose depletion-induced p21 accumulation represents a preemptive step taken by the mother cell to prevent daughter cells from making a harmful choice of proliferation that, as a consequence, would lead to DNA damage.

## STAR★Methods

### Key resources table

**Table:** 

REAGENT or RESOURCE	SOURCE	IDENTIFIER
**Antibodies**		

AMPK	Proteintech	66536-1
phospho-AMPKα (Thr172)	CST	#2535S
p21	CST	#2947S
p53	CST	#2524S
p27	Abclonal	A19095
GAPDH	Abclonal	A19056
CDK2	Abclonal	A0294
phospho-Rb (Ser807/811) conjugated with Alexa Fluor® 488	CST	4277S
phospho-Histone H2A.X(Ser139) conjugated with Alexa Fluor® 488	CST	9719S

**Chemicals, peptides, and recombinant proteins**		

Glucose	Sangon Biotech	A501991
Glutamine	Sangon Biotech	A100374
DMEM/F12 (without glucose, glutamine and phenol, with HEPES)	Procell	PM150329
Propidium iodide (PI)	Sangon Biotech	A601112
RNase A	Sangon Biotech	B600473
Nutlin-3	Selleck	S1061
Indole-3-acetic acid sodium salt (IAA)	Sigma	I5148

**Experimental models: Cell lines**		

HEK293T	ATCC	CRL-3216
hTERT-RPE1	ATCC	CRL-4000
hTERT-RPE1 + Fucci4	This paper	N/A
hTERT-RPE1 + DHB-mVenus + mKO2-Cdt(30-120)-IRES-H1-mMarron1	This paper	N/A
hTERT-RPE1 + p21-miniIAA7-mTurquoise2-NeoR + DHB-mVenus + mKO2-Cdt(30-120)-IRES-H1-mMarron1	This paper	N/A

**Oligonucleotides**		

p21-gRNA: GGAAGCCCTAATCCGCCCAC	This paper	N/A
LHA-F: CTAAAGGGACTAGTCCTGCACTGC CCGCCTTTCTTTTTGAG	This paper	N/A
LHA-R: GGCGCCTGCACCGGATCCGGGCT TCCTCTTGGAGAAGATCA	This paper	N/A
RHA-F: GGTGTTGGGTCGTTGGATCCAGGA AGCCTGCAGTCCTG	This paper	N/A
RHA-R: GCGAATTGAATTTAGCGGCCGCCA CTCAAGGGGGCCTGT	This paper	N/A
miniIAA7-F: CTATAGGGCGAATTGGAGCTCC CCGGGATCCGGTGCAGGCGCCGGCTTCTC TGAGACCGT	This paper	N/A
miniIAA7-R: AGCTCCTCGCCCTTGCTCACGC TAGCGGAGCTTGTCTTCTG	This paper	N/A
mTurquoise2-F: GTGAGCAAGGGCGAGGAGC TGTTCACC	This paper	N/A
mTurquoise2-R: TATGATCAGTTATCTAGATTA CTTGTACAGCTCGTCCATGC	This paper	N/A
negative control sense: UUCUCCGAACGUGUC ACGUTT	This paper	N/A
negative control antisense: ACGUGACACGUU CGGAGAATT	This paper	N/A
p21 siRNA-1 sense: UUCUCCGAACGUGUCACGUTT	This paper	N/A
p21 siRNA-1 antisense: ACGUGACACGUUCGGAGAATT	This paper	N/A
p21 siRNA-2 sense: CCGCGACUGUGAUGCGCUAAUTT	This paper	N/A
p21 siRNA-2 antisense: AUUAGCGCAUCACAGUCGCGGTT	This paper	N/A
p53 siRNA sense: GACUCCAGUGGUAAUCUACTT	This paper	N/A
p53 siRNA antisense: GUAGAUUACCACUGGAGUCTT	This paper	N/A
**Recombinant DNA**		

pLL3.7m-Clover-Geminin(1-110)-IRES-mKO2-Cdt(30-120)	Addgene	Cat #83841
pLL3.7m-mTurquoise2-SLBP(18-126)-IRES-H1-mMaroon1	Addgene	Cat #83842
pSpCas9(BB)-2A-GFP	Addgene	Cat #48138
pSH-EFIRES-B-Seipin-miniIAA7-mEGFP	Addgene	Cat #129719
pMK289	Addgene	Cat #72827
pMK232	Addgene	Cat #72834
FUGW-H1-mMaroon1-IRES-mKO2-Cdt(30-120)	This paper	N/A
pX458-p21-gRNA	This paper	N/A
pBluescript-miniIAA7- mTurquoise2-NeoR	This paper	N/A
FUGW-AtAFB2-mCherry-weak NLS	This paper	N/A

**Software and algorithms**		

TrackMate	Tinevez et al.^18^	http://10.1016/j.ymeth.2016.09.016
R 4.2.2	R	r-project.org

**Deposited data**		

RNA-seq raw data	This paper	BioProject ID: PRJNA861859

**Other**		

objective for time-lapse microscopy imaging	Nikon	CFI S Plan Fluor ELWD 20X C
12-well dish	Thermofisher	#150628

### Resource availability

#### Lead contact

Further information and requests for resources and reagents should be directed to and will be fulfilled by the lead contact, Jun Ma (jun_ma@zju.edu.cn).

#### Materials availability

This study did not generate new unique reagents.

### Experimental model and subject details

#### Cell culture

HEK293T and hTERT-RPE1 cells were maintained in DMEM (Gibco) and DMEM/F-12 (Gibco) media, respectively, supplemented with 10% FBS, penicillin/streptomycin (100 U/ml each) at 5% CO2 and 37°C. For experiments where single nutrients were removed, hTERT-RPE1 cells were washed with PBS at least three times and then cultured in DMEM/F12 medium with 10% dialyzed FBS (BI, 04-011-1A) without phenol and the nutrient of interest. For experiments evaluating different concentrations of glucose limitation, hTERT-RPE1 cells were synchronized to G0 by serum starvation for 24 hr, washed with PBS at least three times and then cultured in DMEM/F12 medium with 1% or 10% dFBS and 0, 0.1, 1, 5 or 17 mM glucose without phenol, respectively.

### Method details

#### Generating Fucci4 hTERT-RPE1 stable cell lines

To package lentivirus, HEK293T cells at ∼70% confluence were transfected with psPAX2, pMD2.G, and pLL3.7m-Clover-Geminin(1-110)-IRES-mKO2-Cdt(30-120) or pLL3.7m-mTurquoise2-SLBP(18-126)-IRES-H1-mMaroon1 plasmids at the ratio of 0.75:0.5:1 using jetPRIME, according to the manufacturer’s instructions (Polyplus). Two days after transfection, supernatant was filtered with a 0.45 μm polyethersulfone filter and then used to infect hTERT-RPE1 cells. After 7 days, positive cells were sorted via flow cytometry.

#### Generating DHB-Cdt(30-120)-H1 hTERT-RPE1 stable cell lines

HEK293T cells at ∼70% confluence were transfected with psPAX2, pMD2.G, and FUGW-DHB-mVenus or FUGW-H1-mMaroon1-IRES-mKO2-Cdt(30-120) at the ratio of 0.75:0.5:1 using jetPRIME, according to the manufacturer’s instructions (Polyplus). Two days after transfection, supernatant was filtered with a 0.45 μm polyethersulfone filter and then used to infect hTERT-RPE1 cells. After 7 days, mVenus and mMaroon1 positive cells were sorted via flow cytometry.

#### Generating p21-miniIAA7- mTurquoise2 hTERT-RPE1 cell lines

The endogenous *CDKN1A* gene was tagged at the C terminus using CRISPR-mediated gene tagging. The p21-gRNA of the CDKN1A stop codon was used. Forward and reverse oligonucleotides for the gRNA were annealed and ligated into BbsI-linearized pSpCas9(BB)-2A-GFP. For the homology donor plasmid, we PCR-amplified *CDKN1A* homology arms from the hTERT-RPE1 genomic DNA using the PrimeSTAR® Max DNA Polymerase (Takara). To PCR-amplify the LHA and RHA, we used LHA-F and LHA-R primers or RHA-F and RHA-R primers, respectively. miniIAA7 cDNA was PCR-amplified from pSH-EFIRES-B-Seipin-miniIAA7-mEGFP with the miniIAA7-F and miniIAA7-R primers. mTurquoise2 cDNA was PCR-amplified from pLL3.7m-mTurquoise2-SLBP(18-126)-IRES-H1-mMaroon1 with the mTurquoise2-F and mTurquoise2-R primers. To generate miniIAA7-mTurquoise2-NeoR plasmid (pBluescript-miniIAA7-mTurquoise2-NeoR), miniIAA7 and mTurquoise2 cDNA were ligated into SacI- and XbaI-cut pMK289 by 3-way ligation using ClonExpress® MultiS One Step Cloning Kit (Vazyme Biotech). miniIAA7-mTurquoise2-NeoR cDNA was isolated as a BamHI fragment from the pBluescript-miniIAA7-mTurquoise2-NeoR vector. LHA, RHA and miniIAA7-mTurquoise2-NeoR cDNA were ligated into SbfI- and NotI-cut pMK232 by 4-way ligation using ClonExpress® MultiS One Step Cloning Kit (Vazyme Biotech). All constructs were determined by sequencing prior to cell transfection.

To generate endogenously tagged p21-miniIAA7-mTurquoise2 cells, the pX458 p21 gRNA plasmid and the p21 homology donor linearized PCR product were transfected into hTERT-RPE1 cells at the ratio of 1:1 using jetPRIME, according to the manufacturer’s instructions (Polyplus). Cells were treated with 400ug/ml G418 until visualized clones generated. Finally, both alleles tagged clones were verified by PCR.

#### Generating AtAFB2 hTERT-RPE1 stable cell lines

HEK293T cells at ∼70% confluence were transfected with psPAX2, pMD2.G, and FUGW-AtAFB2-mCherry-weak NLS at ratio 0.75:0.5:1 using jetPRIME, according to the manufacturer’s instructions (Polyplus). Two days after transfection, supernatant was filtered with a 0.45 μm polyethersulfone filter and then used to infect p21-miniIAA7-mTurquoise2 hTERT-RPE1 cells. After 7 days, mCherry positive cells were sorted via flow cytometry.

#### siRNA transfection

Cells were transfected with 20nM final concentration of siRNA (see Table of key resources table for sequences) using jetPRIME, according to the manufacturer’s instructions.

#### Time-lapse microscopy

Cells were plated 24 hr before imaging in a 12-well dish (Thermofisher). Cells were imaged in a humidified, 37°C chamber in 5% CO_2_. Images were taken in cyan (excitation CWL/BW: 440/10 nm; emission CWL/BW: 460/30 nm), green (490/10 nm; 535/30 nm), red (545/10 nm; 575/25 nm) and cy5 (610/10 nm; 670/65 nm) channels for every 15min on a Nikon Ti2-E inverted microscope (Nikon). Total light exposure time was kept under 500ms for each snapshot. Each channel intensity was no more than 5%.

#### Image analysis

All image analyses were performed with FIJI plugin TrackMate.^18^ For the Fucci4 system, the Histone H1.0-mMaroon1 signal was used to semi-automatically segment different cells on an image for cell identification and tracking in time series. For each cell, a circle with a radius of 5 pixels (0.55μm/pixel) was determined by Laplace of Gaussian filtering (FIJI) as the representative nuclear region, within which the mean pixel intensities of mKO2-Cdt1, Clover-Geminin and mTurquoise2-SLBP were extracted. Optical illumination bias was empirically derived by sampling background areas across all wells in an imaging session and subsequently used to flatten all images. This enabled measurement and subtraction of a global background for each image.

CDK2 activity was calculated as the cytoplasmic to nuclear ratio of CDK2 sensor mean signals. The nuclear signal was measured within the representative region masked by H1.0-mMaroon1. The cytoplasmic signal was measured within the ring with an external diameter of 2 pixels from the nuclear region. CDK2 activity was used to distinguish cells in either a quiescent or a proliferative state. Quiescence was defined when the mean CDK2 activity stayed below 0.8 for more than 10 hr . Otherwise, cells were defined as proliferating. A pair of daughter cells that both underwent quiescence or proliferation after mitosis were defined as a concordant pair. A discordant pair of daughter cells were defined when one was quiescent and the other was proliferative. For the quantification of p21 level, we used the mean reporter intensity within the nuclear region.

#### RNA sequencing

Before library construction, a pellet of ∼100,000 sampled cells were snap-frozen and stored at −80°C. Total RNA was extracted from each sample using RNAiso Plus (Takara). The libraries were sequenced on Illumina NextSeq sequencer with PE150 chemistry. Each experiment had two biological replicates.

Overall sequence quality was examined using FastQC v0.11.2. Adaptor sequences were clipped using Trimmomatic v0.32.^38^ Reads were mapped to the human genome (GRCh38) using HISAT2 v2.0.3-beta.^39^ Mapped fragments were counted using featureCounts.^40^ Differential expression analysis was carried out using DESeq2 v1.10.1.^41^ Genes with an adjusted p value less than 0.05 were considered to be differentially expressed. GO analysis was carried out using clusterProfiler.^42^

#### Western blotting

Cells were collected and then directly lysed by addition of RIPA buffer (FUDE, FD009). Whole cell lysates were loaded onto 12% SDS-PAGE (FUDE, FD346) followed by transfer to PVDF membranes (Millipore, IPVH00010). After protein transfer, membranes were incubated in 5% milk in TBST at room temperature (RT) with rocking for at least 2 hr. Primary antibody diluted in 5% milk in TBST was added and membranes were incubated overnight at 4°C with rocking. Membranes were washed three times in TBST and anti-mouse or anti-rabbit HRP-conjugated secondary antibodies (FUDE) were diluted 1:10,000 in 5% milk in TBST and incubated with membranes at RT with rocking for 1 hr. Membranes were washed three times in TBST and visualized using ECL Western Blotting Substrate (Vazyme Biotech, E412-01). Blots were scanned using a gel imaging system and the image was analyzed by Gel-Pro analyzer software (v4.0.0.001). Antibodies used in this study for Western blotting are shown in table of key resources.

#### Coimmunoprecipitation

Immunoprecipitations of p21 from hTERT-RPE1 cells (control) and p21-miniIAA7-mTurquoise2 hTERT-RPE1 cells were performed using the protein A/G magnetic bead (Abclonal, RM02915), according to the manufacturer’s instructions. Eluted samples were separated by SDS-PAGE, transferred to PVDF membrane and probed with anti-p21 and anti-CDK2 antibodies.

#### Apoptosis assay

Quantitation of apoptotic cells was performed using the Annexin V-FITC detection kit (Beyotime) according to the manufacturer’s protocol. Briefly, cells were cultured on a 6-well dish at the density of 1× 10^6^ cells/well. Following treatment, cells were collected, washed with PBS and re-suspended in 195 μL binding buffer containing 5 μL Annexin V-FITC and 10 μL PI, followed by incubation for 15 min at room temperature in the dark. Finally, fluorescence was quantified by flow cytometry, where apoptotic cells were defined as positive staining for Annexin V or PI.

#### Flow cytometry

Cells were fixed with 4% paraformaldehyde at 4°C for 30 min and washed three times with PBS, followed by permeabilization in 0.1% Triton X-100 for 10 min. Cells were then incubated with primary antibodies, phospho-Rb (Ser807/811) conjugated with Alexa Fluor® 488 and phospho-Histone H2A.X (Ser139) conjugated with Alexa Fluor® 488, at 4°C for 1 hr. After washing three times with 5% bovine serum albumin protein (BSA), cells were incubated with 50 mg/ml RNase A and 40 mg/ml propidium iodide (PI) for 30 min. Identification of cell cycle phases was achieved through flow cytometry analysis.

### Quantification and statistical analysis

Quantified results were reported as mean ± SD unless specified otherwise. Statistical analysis was performed using the unpaired, two-tailed Student’s t-test contained and one-way ANOVA followed by Dunnett’s multiple comparisons test in the GraphPad Prism v9.0.0 software. Significance levels are indicated in figure legends. Coefficient of determination (*R*^2^) to measure the difference between sibling cells was computed as $2\sum_{i}{\left(x_{i}-y_{i}\right)}^{2}/\left(\sum_{i}{\left(x_{i}-\bar{x}\right)}^{2}+\sum_{i}{\left(y_{i}-\bar{y}\right)}^{2}\right)$, in which *x*_*i*_ and *y*_*i*_ denote the p21 levels of a non-ordered sibling pair.

## Data Availability

• RNA sequence raw data is deposited at SRA (http://www.ncbi.nlm.nih.gov/sra) with the BioProject ID: PRJNA861859. • RNA-seq analyses were performed using standard software packages. Code for live imaging data analysis is available upon request. • Any additional information required to reanalyze the data reported in this paper is available from the lead contact upon request.
