# Supplementary material for: Daughter cell fate choice instructed preemptively by mother cells facing nutrient limitation
Source: iScience. 2023 Jun 24;26(7):107198. doi: 10.1016/j.isci.2023.107198 (PMC10359942; doi:10.1016/j.isci.2023.107198)
Supplement: Document S1. Figures S1–S8 [file mmc1.pdf]

iScience, Volume *26*

Supplemental information

Daughter cell fate choice instructed preemptively by mother cells facing nutrient limitation

Dianpeng Zheng, Yaowen Mao, Yinglong Gao, Feng He, and Jun Ma

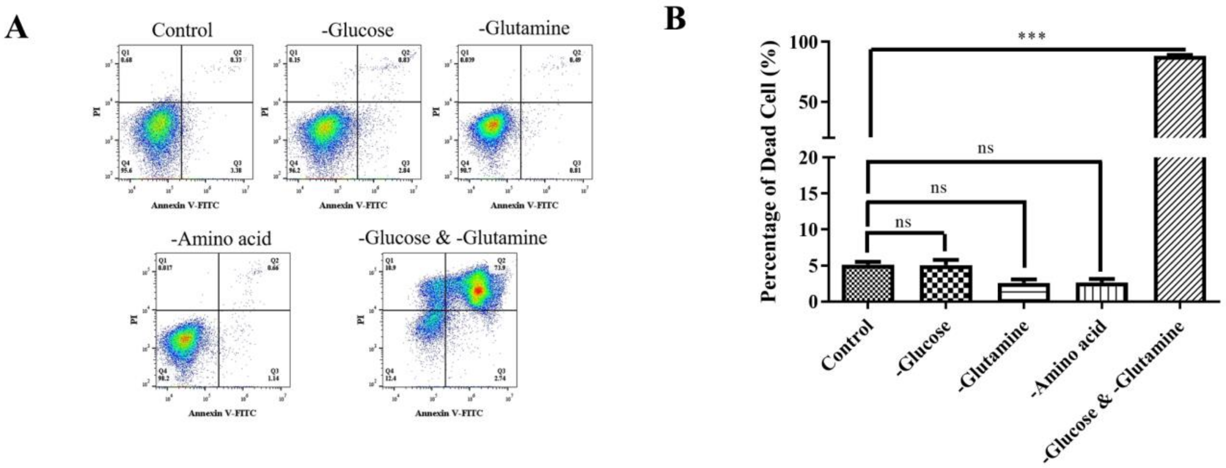

Figure S1. Cells deprived of a single nutrient were mostly viable, Related to Figure 1. (A) Analysis of cell death by flow cytometry for hTERT-RPE1 cells under deprivation of amino acids, glucose, glutamine, or both glucose and glutamine for 48 hr. Annexin V FITC and PI were used for staining. (B) Percentages of dead cells quantified from Q1 + Q2 + Q3 in panel (A). Error bars represent standard deviations of three independent experiments. *** and "ns" represent *p*-values of Student's t-tests $< 10^{-3}$ or > 0.05, respectively.

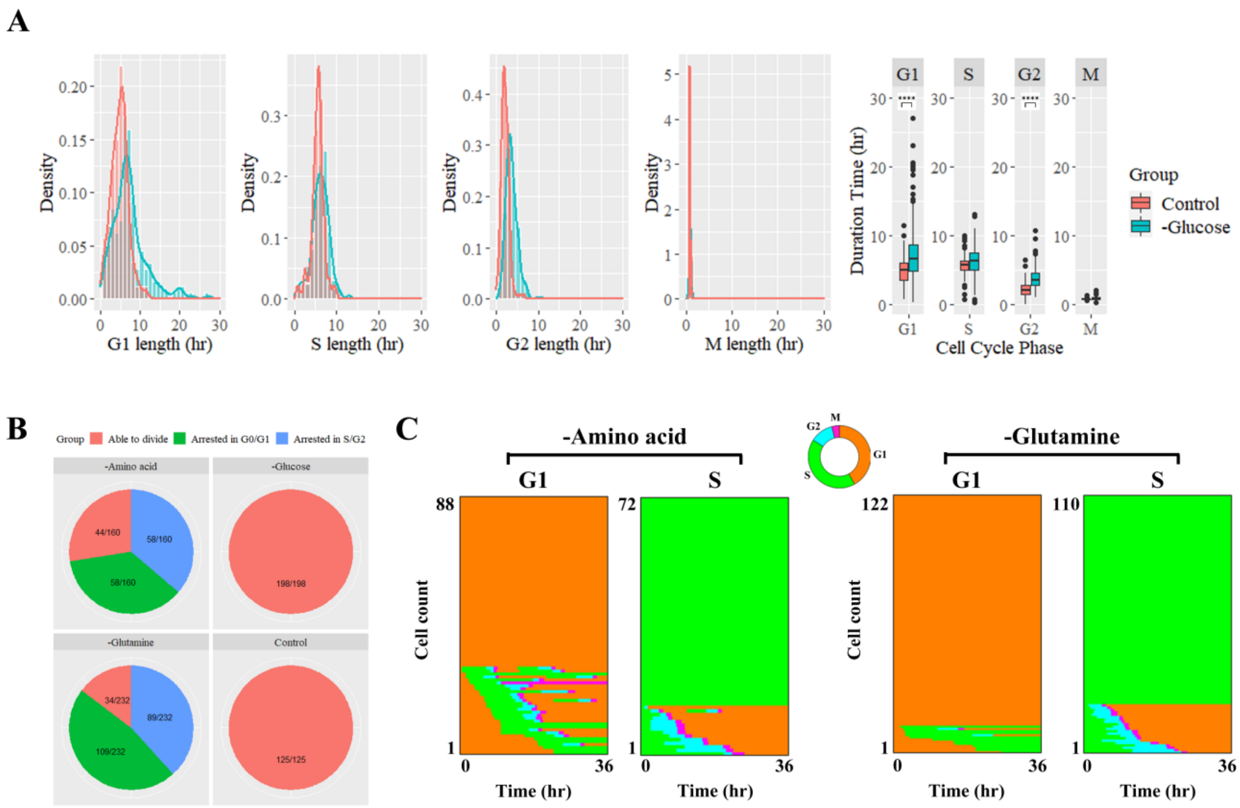

Figure S2. Glucose-deprived cells were able to slowly proliferate, Related to Figure 1. (A) Glucose-deprived cells had elongated G1 and G2 phases. **** represent *p*-values of Student's t-tests < 10^{-4}. (B) Shown are pie charts of the total observed cell counts and those that successfully divided, arrested in G0/G1, or arrested in S/G2 under different nutrient depletion conditions. (C) Same as Figure 1C but for cells without amino acids or glutamine.

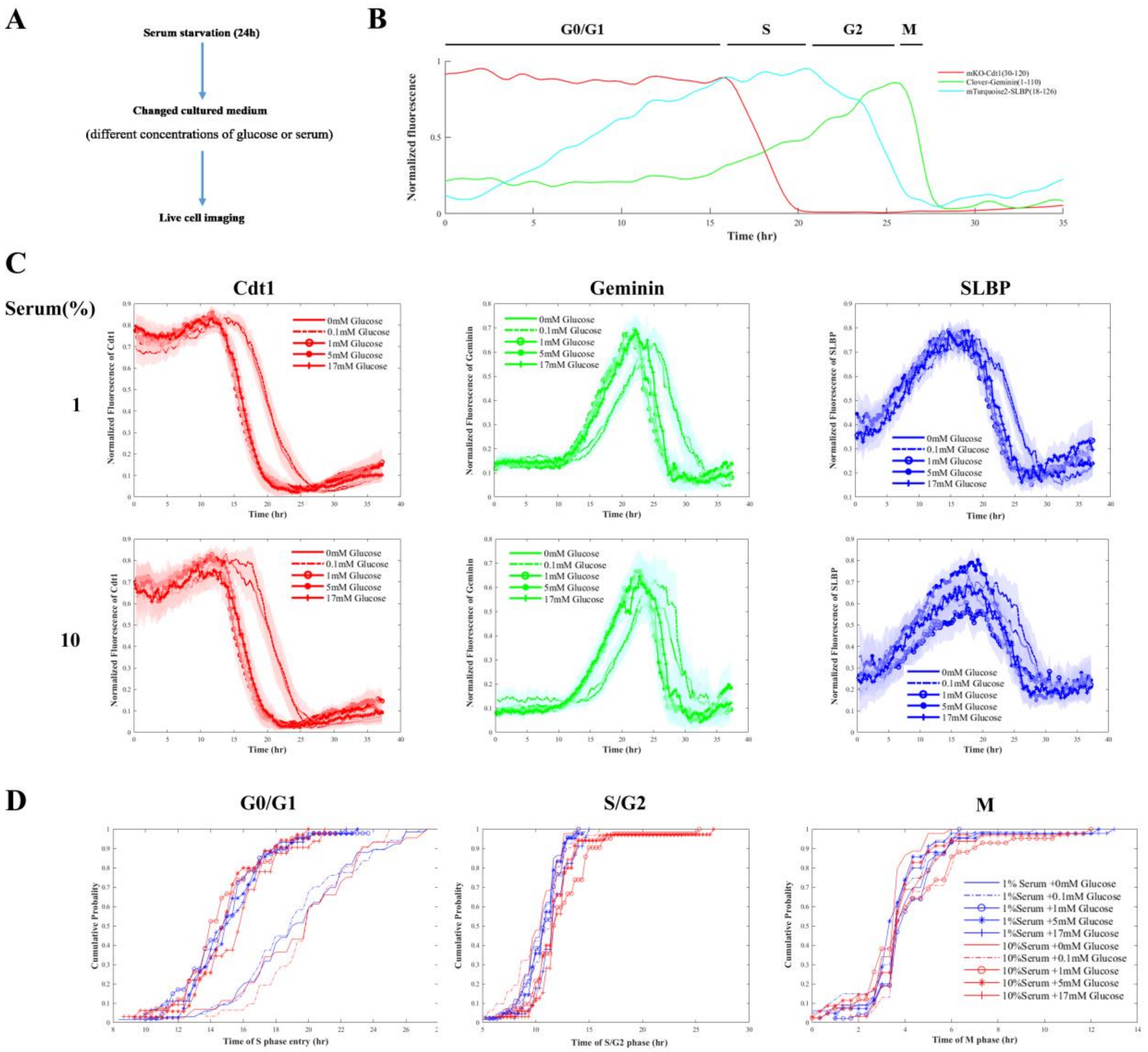

Figure S3. A live imaging analysis of individual cells synchronized in G0/G1 by serum starvation, Related to Figure 1. (A) A schematic diagram of the experimental design to evaluate the effect of glucose limitation to cells that were synchronized in G0/G1 using our hTERT-RPE1 Fucci4 reporter system. (B) Fucci4 intensity profiles (red: Cdt1, green: Geminin, blue: SLBP) of a representative cell from a population that were synchronized in G0/G1 by serum starvation (DMEM/F-12 without serum supplement) for 24 hr and then resupplied with a serum-containing medium (DMEM/F-12 with 10% serum supplement). DMEM/F-12 contains glucose at a concentration of 17 mM. Time 0 was the time of medium replacement. (C) Mean intensity profiles of Fucci4 reporters of a synchronized population of cells after different medium replacements. Ten combinations of serum (s) and glucose (g) concentrations were tested: 1% s + 0 g (N = 25, solid lines in upper panels), 1% s + 0.1 mM g (N = 50, dashed lines in upper panels), 1% s + 1 mM g (N = 66, open circles in upper panels), 1% s + 5 mM g (N = 48 filled circles in upper panels), 1% s + 17 mM g (N = 41, filled bars in upper panels), 10% s + 0 g (N = 22, solid lines in lower panels), 10% s + 0.1 mM g (N = 32, dashed lines in lower panels), 10% s+ 1 mM g (N = 27, open circles in lower panels), 10% s + 5 mM g (N = 26 filled circles in lower panels), and 10% s + 17 mM g (N = 23, filled bars in lower panels). The shades represent 95% confidence intervals. In this experiment, the measured G0/G1 duration lengths (in 10% serum concentration) were 25.56 ± 6.41, 25.78 ± 4.49, 21.11 ± 4.62, 19.56 ± 4.23 and 19.78 ± 4.87 hr at 0, 0.1, 1, 5 and 17mM glucose, respectively. These results revealed that G0/G1 lengthening took place between 0.1 and 1mM glucose. Importantly, this responding range of glucose concentration is identical to that in the quiescence-proliferation choice response (Figure2D; Supplemental Figure 4C) and molecular responses (Figure 3D). (D) Empirical cumulative distribution function plots of the measured durations of G0/G1 (the time of serum supplement to S phase entry), S/G2 (S phase entry to M phase entry) and M (M phase entry to completion of division) for individual cells under the tested conditions (see Materials and Methods for defining different cell cycle stages). Discussion of Figure S3. The measured G0/G1 duration lengths were 25.56 ± 6.41, 25.78 ± 4.49, 21.11 ± 4.62, 19.56 ± 4.23 and 19.78 ± 4.87 hr at 0, 0.1, 1, 5 and 17 mM glucose, respectively. Here, G0/G1 lengthening took place between 0.1 and 1 mM glucose, documenting the same responding range of glucose limitation in cell cycle response.

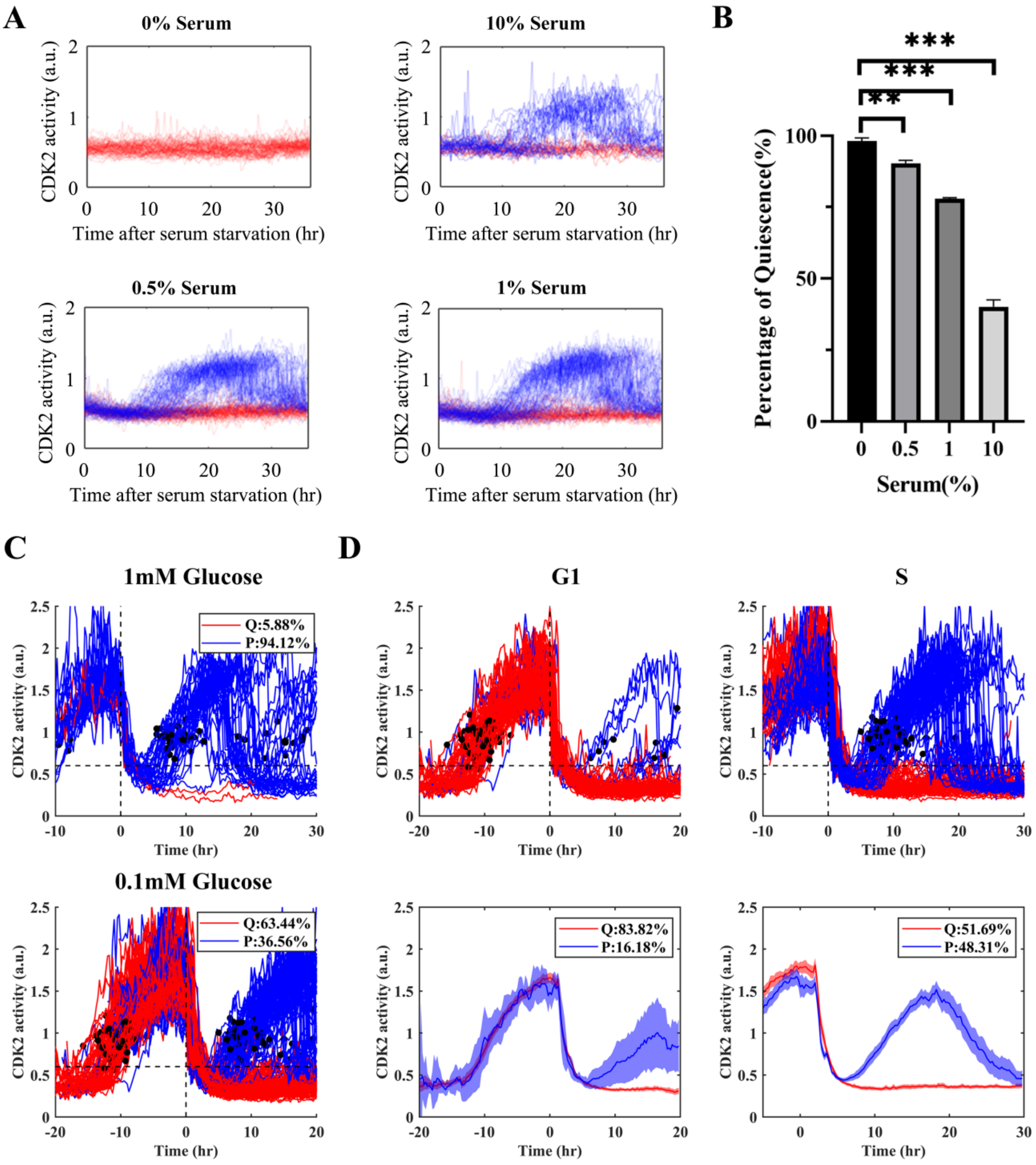

Figure S4. Response of CDK2 activity to serum and glucose limitations in individual cells, Related to Figure 2. (A) Dynamic profiles of CDK2 activity measured from hTERT-RPE1 cells stably expressing a CDK2 activity sensor. Cells were synchronized by serum starvation for 24 hr, and then replenished with 0 (N = 80), 0.5% (N = 107), 1% (N = 105) and 10% (N = 33) serum, respectively. Cell fates were determined as described in Figure 2A (red: quiescence; blue: proliferation). (B) Percentages of quiescent cells measured from panel (A). Error bars represent standard errors of the mean. ** and *** represent *p*-values of Student's t-tests = 0.01 and < 10^{-3}, respectively. (C) Same as Figure 2D but for cells cultured in media containing 1 mM (N = 34; upper) or 0.1 mM glucose (N = 351; lower). (D) Same as Figure 2E but for cells cultured in media containing 0.1 mM glucose.

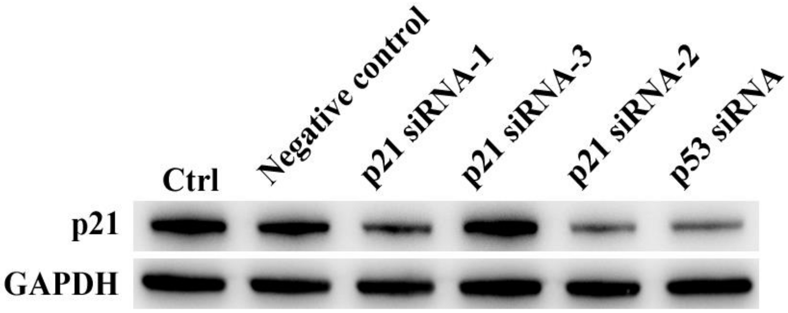

Figure S5. Shown are Western blotting results detecting p21 protein under different siRNA treatments**, Related to Figure 3**. hTERT-RPE1 cells were transfected with a negative control siRNA, three p21 siRNAs and a p53 siRNA for 48 hr and then detected p21 level by Western blot. GAPDH levels was used as an internal control. As shown in Figure 3, p21 siRNA-3 was excluded from our flow cytometry experiments due to its inability in reducing p21 protein level.

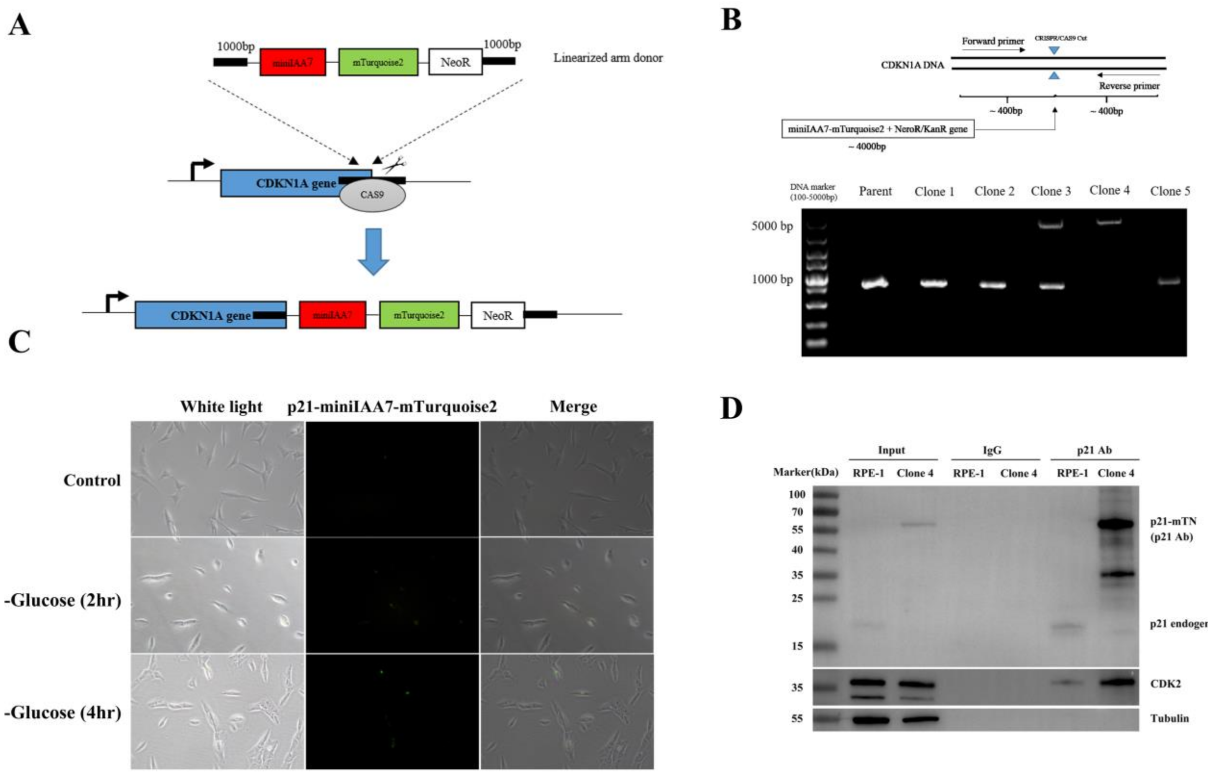

Figure S6. Construction of a p21 fusion reporter for live imaging, Related to Figure 4. (A) A schematic diagram depicting the construction of an endogenous reporter p21-miniIAA7-mTurquoise2. Primers were antisense to ~1000 bps upstream and ~1000 bps downstream of the CRISPR cutting site (black triangles). The length of inserted fragments (miniIAA7-mTurquoises and NeroR/KanR) was ~4000 bps, with the final product containing an insertion expected to be ~5000 bps. (B) Selection of clones with positive PCR products. Clone 4 was correctly tagged at both alleles. Clone 3 was tagged at only one allele. Other tested clones had no correct insertion. Parent: hTERT-RPE1 cell line. (C) Representative images showing the fluorescent intensities of p21-miniIAA7-mTurquoise2 under glucose deprivation for 0, 2 and 4 hr, respectively. (D) Coimmunoprecipitation of CDK2 with p21 in Clone 4 and hTERT-RPE1 cell line. Immunoprecipitation was performed using antibodies against p21. Western blotting was performed to detect p21 and CDK2. p21-miniIAA7-mTurquoise2 (p21-mTN) was detected with an expected size of ~60 kDa.

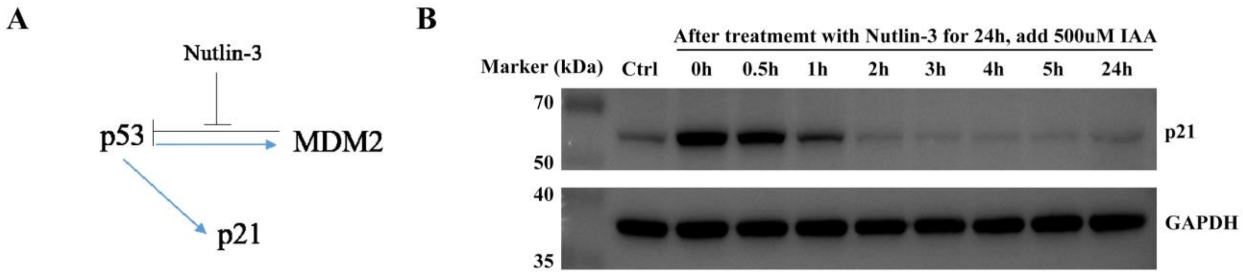

Figure S7. AID system is suitable for rapidly degradation of endogenously expressed p21 fusion protein, Related to Figure 6. (A) A simplified regulatory network in response to Nutlin-3. (B) Cells were treated with 10 μM Nutlin-3 for 24 hr, followed by addition of 500 μM IAA. Shown are Western blotting results detecting the p21 fusion protein and an internal control (GAPDH) at the indicated time after IAA addition.

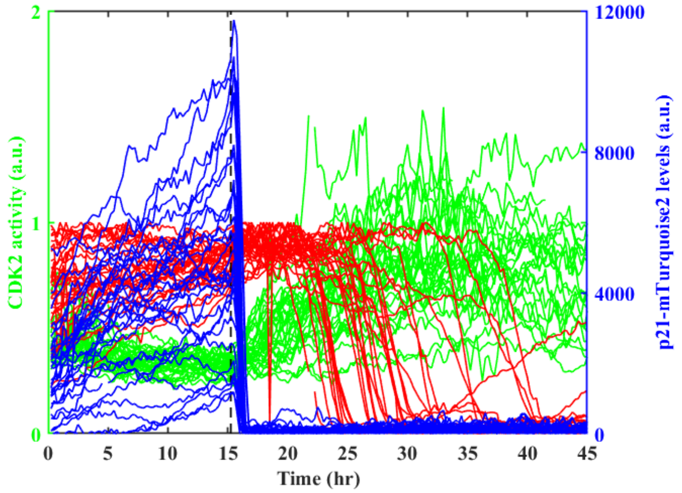

Figure S8. Time-lapse imaging of cells with the p21 AID system, Related to Figure 6. Quantitative profiles of cells with the p21 AID system under sustained glucose depletion and with induced p21 degradation as described in Figure 6D. Green: CDK2 activity; red: Cdt1 intensity (normalized to 0-1); blue: p21 intensity.
